# Coordination of Chromosome Segregation and Cell Division in *Staphylococcus aureus*

**DOI:** 10.3389/fmicb.2017.01575

**Published:** 2017-08-23

**Authors:** Amy L. Bottomley, Andrew T. F. Liew, Kennardy D. Kusuma, Elizabeth Peterson, Lisa Seidel, Simon J. Foster, Elizabeth J. Harry

**Affiliations:** ^1^The ithree Institute, University of Technology Sydney, Sydney NSW, Australia; ^2^Department of Molecular Biology and Biotechnology, Krebs Institute, University of Sheffield Sheffield, United Kingdom

**Keywords:** cell division, chromosome segregation, protein–protein interactions, divisome, *Staphylococcus aureus*, DivIVA, DnaK

## Abstract

Productive bacterial cell division and survival of progeny requires tight coordination between chromosome segregation and cell division to ensure equal partitioning of DNA. Unlike rod-shaped bacteria that undergo division in one plane, the coccoid human pathogen *Staphylococcus aureus* divides in three successive orthogonal planes, which requires a different spatial control compared to rod-shaped cells. To gain a better understanding of how this coordination between chromosome segregation and cell division is regulated in *S. aureus*, we investigated proteins that associate with FtsZ and the divisome. We found that DnaK, a well-known chaperone, interacts with FtsZ, EzrA and DivIVA, and is required for DivIVA stability. Unlike in several rod shaped organisms, DivIVA in *S. aureus* associates with several components of the divisome, as well as the chromosome segregation protein, SMC. This data, combined with phenotypic analysis of mutants, suggests a novel role for *S. aureus* DivIVA in ensuring cell division and chromosome segregation are coordinated.

## Introduction

Cell division is an essential process for the propagation of all living species. In most bacteria, the first stage of cell division involves the polymerization of FtsZ into a dynamic structure at midcell called the Z-ring (Lutkenhaus et al., 2012; Busiek and Margolin, 2015; Bisson-Filho et al., 2017; Yang et al., 2017). The Z-ring provides part of the contractile force for the invagination of the cell envelope (Osawa et al., 2008; Erickson et al., 2010; Daley et al., 2016) and acts as a scaffold for the recruitment of a set of at least 12 different proteins which, through a web of protein–protein interactions, form a large protein complex at the division site (Adams and Errington, 2009; Rico et al., 2013). This protein complex, known as the divisome, catalyzes the formation of the septal crosswall resulting in progeny with the correct genetic content.

Spatial and temporal regulation of the cell division process must be coupled to chromosome replication and segregation to ensure equal partitioning of DNA into two newborn cells. Z-ring assembly is therefore finely regulated to avoid guillotining of the nucleoid by the division septum. Both positive (Willemse et al., 2011; Treuner-Lange et al., 2013; Fleurie et al., 2014) and negative regulators (see below for references) of Z-ring placement have been described in a number of bacterial species. The best characterized of these regulation systems are nucleoid occlusion and the Min system that act negatively in Z-ring assembly. Nucleoid occlusion prevents Z-ring formation over replicating chromosomes via the Noc protein in *Bacillus subtilis* and *Staphylococcus aureus* (SlmA in *Escherichia coli*; Bernhardt and De Boer, 2005) (Wu and Errington, 2004; Veiga et al., 2011). Many bacterial species also encode Min proteins, and in *B. subtilis* these comprise: MinC, which physically inhibits Z-ring formation; MinD, a Walker-type ATPase; MinJ that acts as a bridging protein between MinD and the membrane-associated DivIVA, which accumulates at negative-curved membranes to localize the Min complex (Bi and Lutkenhaus, 1990; de Boer et al., 1991; Varley and Stewart, 1992; Lee and Price, 1993; Bramkamp et al., 2008; Patrick and Kearns, 2008; Lenarcic et al., 2009; Ramamurthi and Losick, 2009). The Min system was originally thought to be stably attached to the cell pole regions via DivIVA to prevent Z-ring formation at these sites, but it has been more recently shown that DivIVA dynamically relocates to active septa, suggesting that the Min system exerts its effect at the septum rather than cell poles, at least in *B. subtilis* (Gregory et al., 2008; van Baarle and Bramkamp, 2010; Bach et al., 2014).

A link between chromosome segregation and cell division has been observed in several bacteria, including *B. subtilis* and *Caulobacter crescentus*, where the regulation of Z-ring placement and formation is in part carried out by chromosome segregation and organization proteins. Absence of these proteins (e.g., SMC; ParB and homologs) gives rise to misplaced Z-rings, resulting in anucleate minicells, or a block in cell division to produce filamentous cells (Ireton et al., 1994; Britton et al., 1998; Mohl et al., 2001). Additionally, DivIVA, which is classically described as part of the Min system, has also been shown to have a role(s) independent of Min proteins, where is has been implicated in chromosome segregation in a variety of bacterial species including *Enterococcus faecalis*, *B. subtilis*, and *Streptococcus pneumoniae* (Ben-Yehuda et al., 2003; Ramirez-Arcos et al., 2005; Fadda et al., 2007).

The human pathogen *S. aureus* is coccoid in shape and divides in three consecutive orthogonal planes (Tzagoloff and Novick, 1977; Turner et al., 2010). *S. aureus* does not have the Min system, raising the question of how Z-ring placement is coordinated within the cell cycle. The mode of division site placement in *S. aureus* is not established but has been proposed to involve a role for Noc and an epigenetic inheritance of cell wall architectural features (Turner et al., 2010; Veiga et al., 2011). The *S. aureus* genome encodes homologues of several components of the *B. subtilis* chromosome partitioning machinery such as Spo0J (ParB), FtsK, SpoIIIE and SMC (Yu et al., 2010; Veiga and Pinho, 2017) but does not contain a Soj (ParA) homolog (Pinho et al., 2013). Furthermore, despite the absence of the Min system, a DivIVA homolog is present in *S. aureus*, although without a defined function (Pinho and Errington, 2004).

To gain an understanding of how chromosome segregation is coordinated with cell division in *S. aureus*, we sought to understand how these processes might be linked. We identified a range of novel interactions that reveal how cell division proteins may facilitate chromosome segregation. Our data further suggests a(n) (indirect) role for DivIVA, together with the chromosome partitioning protein, SMC, in maintaining accurate chromosome segregation in this organism.

## Results

### Identification of Novel Protein Interactions with Known *S. aureus* Divisome Components

**FIGURE 1:**
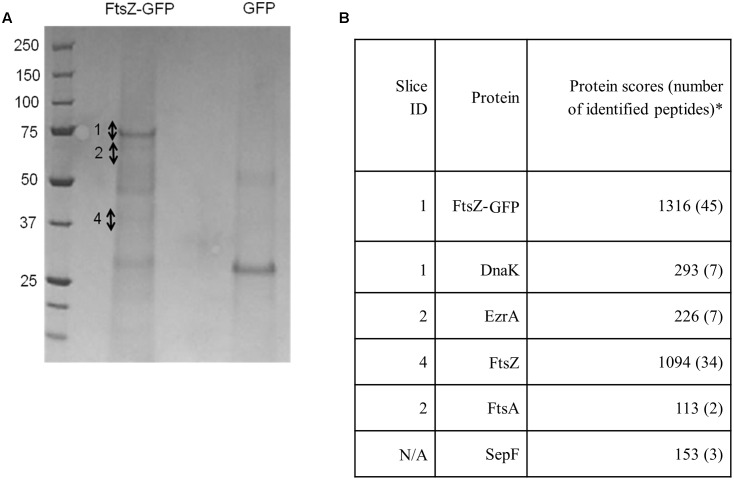
FtsZ, EzrA, FtsA, SepF and DnaK are associated with FtsZ-GFP *in vivo* in *S. aureus*. Coomassie-stained SDS-PAGE gel **(A)** with corresponding table **(B)** showing significant protein hits unique to the FtsZ-GFP sample identified from gel slices. Numbers beside the Coomassie-stained bands in the ‘FtsZ-GFP’ lane indicate individual gel slices that were excised for LC-MS-MS. SepF was not identified from individual bands but was instead obtained from digestion of the whole gel lane. It should be noted that whilst FtsZ was detected predominantly in slice 4, it was also detected to a lesser extent in a number of other slices, likely due to contamination because of the high abundance of FtsZ protein in the sample. The asterisk denotes proteins presented in this study that are identified as “significant hits” and contain at least two peptides with a Mascot score of >100 from two independent experiments (Cooper et al., 2009). Proteins that were present in both the FtsZ-GFP sample and the GFP control sample were considered to be non-specific interacting proteins and were excluded from further analysis.

To identify novel proteins that interact with *S. aureus* FtsZ or FtsZ-associated proteins *in vivo* we utilized a previously described GFP affinity-purification strategy (Cristea et al., 2005) using a *S. aureus* strain that expresses *ftsZ-gfp* from plasmid pLOW (*spa^-^* pLOW-*ftsZ-gfp* pGL485; SA103). Expression of *ftsZ-gfp* was induced with 0.05 mM IPTG as this level of induction has been previously shown to have no observable effect on cellular morphology, cell division or Z-ring formation (Liew et al., 2011). Protein complexes were isolated (see Experimental Procedures for details), separated by SDS-PAGE and bands that were clearly visible by Coomassie staining, as well as remaining gel fragments, were excised and analyzed by liquid chromatography-tandem mass spectrometry (LC-MS-MS; **Figure 1**). A *S. aureus* strain expressing only GFP from pLOW (*spa^-^* pLOW*-gfp* pGL485; SA112) was used as a control to identify proteins not associated specifically with FtsZ, and these were excluded from analysis. Four known division proteins were identified: FtsZ, EzrA, FtsA and SepF. The identification of the self-polymerizing FtsZ protein was expected. EzrA is a known FtsZ-interacting division protein in *B. subtilis* (Ishikawa et al., 2006) and in *S. aureus* interacts with FtsZ in a bacterial two-hybrid assay (Steele et al., 2011). SepF and FtsA have also been previously shown to associate with other Staphylococcal cell division proteins using bacterial two-hybrid assays (Steele et al., 2011). In *B. subtilis*, SepF and FtsA co-purify with FtsZ from *in vivo* cell extracts (Jensen et al., 2005; Ishikawa et al., 2006). Thus, our results demonstrates the successful isolation of *S. aureus* division components using GFP-based affinity purification, and provides further evidence that EzrA, SepF and FtsA proteins are genuinely part of an FtsZ-containing complex in *S. aureus in vivo*.

DnaK was also detected using the FtsZ-GFP pulldown approach. This was of interest as it has been previously observed to be associated with FtsZ in *E. coli* (Calloni et al., 2012). DnaK is highly conserved in bacteria where it serves as a molecular chaperone during heat stress (Bukau and Horwich, 1998). This protein has not previously been described as being associated with the divisome in *S. aureus* and localization of DnaK-msfGFP (monomeric superfolder-GFP: Landgraf *et al., 2*012) using a plasmid-based system (pLOW-*dnaK-msfgfp* pGL485; SA307) showed a uniform signal throughout the cell cytoplasm with no septal-specific localization pattern (Supplementary Figure 1). This cytoplasmic localization pattern likely reflects interaction of DnaK with various cytoplasmic proteins (Calloni et al., 2012), not just those associated with the divisome.

Although DnaK did not show specific localization to midcell, the potential interaction between DnaK and other divisome components was investigated using the bacterial two-hybrid system (Karimova et al., 1998) anticipating that this might reveal more divisome interactions. Consistent with the affinity-purification results (**Figure 1**), a direct interaction was detected between DnaK/EzrA, and DnaK/FtsZ (Supplementary Figures 2A,C). Furthermore a novel direct interaction between DnaK/DivIVA was identified. DivIVA has been shown to localize to the division site in *S. aureus* but its role is unclear (Pinho and Errington, 2004). We therefore further investigated the possible involvement of DivIVA in *S. aureus* cell division.

### DivIVA Is Part of the *S. aureus* Divisome

Detection of an interaction between DnaK and DivIVA led us to investigate the interaction profile of DivIVA with *S. aureus* divisome components using the bacterial two-hybrid assay. We detected several pair-wise interactions with DivIVA including FtsZ, EzrA, FtsA, DivIC, DivIB, PBP1 and PBP2 (Supplementary Figures 2B,D).

**FIGURE 2:**
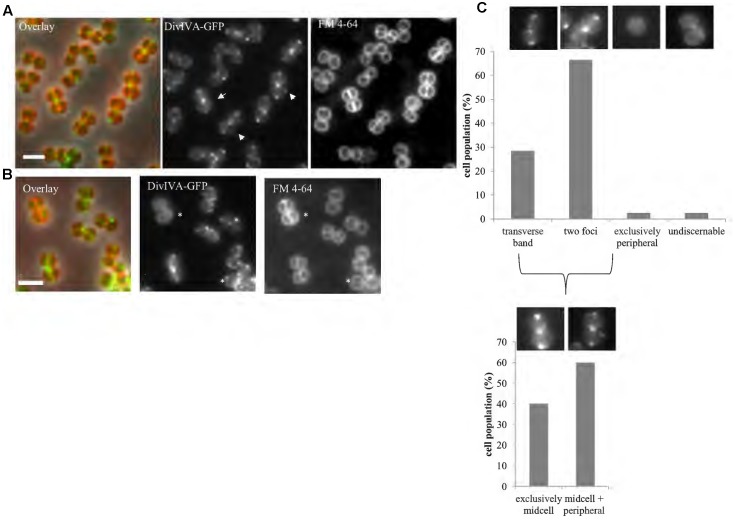
DivIVA localizes to the division site and cell periphery. Representative images of cells expressing a single copy of *divIVA-gfp* (**A**; SA247 [P*_divIV A_ divIVA-gfp*::P*_spac_ divIVA*, pGL485]). White arrows indicate cells which show both localization of DivIVA-GFP at septal membranes while also forming foci or arcs at the non-septal cell periphery. A small percentage of cells (∼2%) show only non-septal localization of DivIVA-GFP as indicated by an asterisk in **(B)**. Live cell wide-field microscopy was performed by growing SA247 cells in BHI broth at 37°C to mid exponential phase, staining with FM 4-64 to identify septal membranes and visualized on 2% agarose pads. Scale bar represents 2 μm. **(C)** Histograms showing the frequency of observed DivIVA-GFP localization patterns, with example microscopic images of DivIVA-GFP localization patterns shown above the corresponding phenotypic category (*n* > 100 cells from 2 independent experiments). Analysis was limited to cells that showed visible septal FM 4-64 staining to exclude cells in which the septa were oriented in the *x-y* (lateral) plane. Analysis of cells showing septal DivIVA-GFP localization (either as a transverse band or two foci) showed that a subpopulation also displayed additional peripheral localization, as indicated in the bottom histogram and images.

Using a functional DivIVA-GFP fusion (Supplementary Figure 3), we confirmed midcell localization for this protein (**Figure 2**) as has been previously shown (Pinho and Errington, 2004). Further analysis of DivIVA-GFP co-localization with the septal membrane (stained with FM4-64) revealed that 60% of cells displayed additional non-septal localization (**Figure 2**), including foci or arcs that are perpendicular to the septal rings and a small percentage of cells (∼2%) which showed exclusive localization of DivIVA-GFP to the cell periphery (**Figure 2**).

### A Function for DivIVA and DnaK in *S. aureus*

A previous study to investigate the physiological role of DivIVA in *S. aureus* using a markerless deletion of *divIVA* showed no obvious phenotype with regards to growth rates and gross chromosome morphology (Pinho and Errington, 2004). We confirmed these results and also found no observable phenotype using a markerless *divIVA* deletion in SH1000 (*spa^-^* Δ*divIVA*; SA365; data not shown – see Experimental Procedures for more detail).

As DivIVA and DnaK interact (Supplementary Figure 2), the likely relationship between the two proteins was investigated. We found that deletion of both genes led to an exacerbation of the anucleate phenotype exhibited by the *dnaK* single mutant. We detail our phenotypic analysis of both single and double mutants below.

**FIGURE 3:**
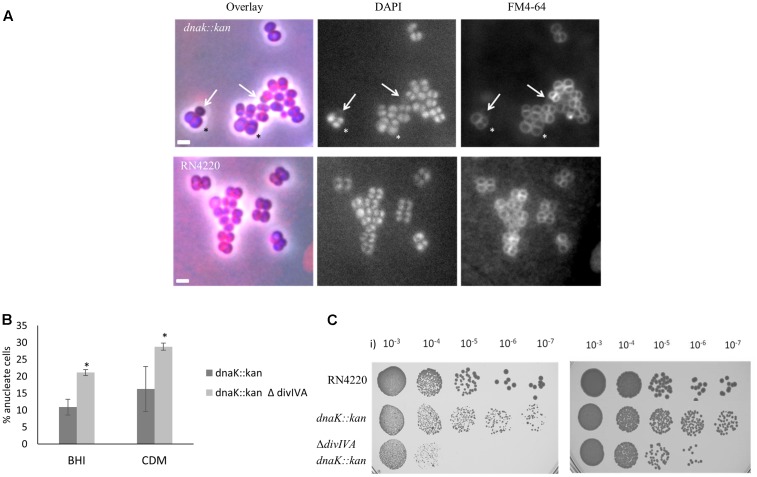
Loss of *dnaK* and *divIVA* results in anucleate cells and decreased cell viability. Overlay of DAPI and FM 4-64 fluorescence images of the *S. aureus dnaK::kan* insertion mutant (SA210) cells (**A**; top panel) and isogenic RN4220 cells (bottom panel). Arrows indicate anucleate cells with faint FM 4-64 staining. The asterisk indicates cells with enlarged diameters. Cells were grown in BHI broth at 37°C to mid-exponential phase of growth and visualized on 2% agarose pads. Anucleate cells were scored based on the absence of DAPI staining. Average percentages of anucleate cells in the population in the *dnaK::kan* single mutant (SA210) compared to the Δ*divIVA dnaK::kan* double mutant (SA213) when grown in rich media (BHI) or amino acid-limiting chemically defined media (CDM) when grown at 37°C **(B)**. Error bars (95% CIs) and averages were calculated from *n* = 230 cells from two independent experiments. Error bars represent 95% CIs. The asterisk indicates that the difference in anucleate cell number is significant between the single and double mutant using the Fisher’s exact test (*P*-value < 0.05). Viability of stationary phase culture of SA210 and SA213 cells were compared to RN4220 wild-type cells in CDM (i) and BHI (ii) media **(C)**. Numbers indicate dilutions used for plating. Cell biomass was normalized using OD_600_ values.

An *S. aureus dnaK::kan* insertion mutant (SA210) showed a significant decrease in growth rate (**Table 1**) at 37°C compared to *S. aureus* RN4220, consistent with previous observations obtained with a *S. aureus* COL *dnaK::kan* insertion mutant (Singh et al., 2007). However, contrary to the previous study, we found that the *dnaK::kan* insertion mutant had a significantly slower growth rate when also grown at 30°C (29 min for RN4220 vs. 47 min in the *dnaK::kan* insertion mutant). This discrepancy is possibly due to differences in media types and strain backgrounds in each study. Examination of *dnaK::kan* cells by microscopic analysis when grown in BHI at 37°C (**Figure 3A** and **Table 1**) showed that there was a 22% increase in average cell diameter compared to wild-type (1.1 ± 0.03 μm compared to 0.9 ± 0.02 μm) suggestive of a cell division defect in the *dnaK::kan* cell population. In addition to this, a significant proportion of cells in the population (10% vs. 0% for RN4220; **Table 1**) were anucleate. A similar frequency of anucleate cells was also observed in the *dnaK* mutant when grown at 30°C (**Table 1**). Importantly, both these morphological changes were not due to a polar effect of the *dnaK::kan* insertion because ectopic expression of *dnaK* from pLOW led to a reduction in both cell size and the frequency of anucleate cells back to levels resembling those of wild-type cells (Supplementary Figure 4). Thus, absence of *dnaK* results in anucleate cells in 10% of the cell population under non-heat stressed conditions.

**Table 1:** Growth rate and percentage of anucleate cells of RN4220 single and double mutants in BHI.

Strain	Temperature (°C)	% Anucleate cells^∗^	Average cell diameter in μm^§^ (% increase)	Growth rate¶ (minutes)
RN4220	30	0	0.86 ± 0.02	51 ± 3
	37	0	0.9 ± 0.02	30 ± 3
SA210	30	11 ± 1	0.97 ± 0.02	65 ± 4
			**(13%)**	
	37	10 ± 2	1.1 0.03	46 ± 4
			**(22%)**	
SA167	37	0	0.90 ± 0.02	27 ± 2
SA213	37	21.1 + 0.3	1.05 ± 0.05	43 ± 2
			**(17%)**	
SA266	37	1.85 ± 0.35	0.99 ± 0.01	37 ± 0
			**(9%)**	
SA269	37	6.4 ± 0.2	0.93 ± 0	41 ± 2
			**(2%)**	


*Standard deviation of percentage of anucleate cells and cell size was obtained from scoring *n* = 200 cells from two independent experiments. Standard error of the mean of the percentage of anucleate cells (^∗^), average cell diameter (§) or growth rate (¶) from two independent experiments is shown as indicated. Strain genotypes are: SA210 (RN4220 *dnaK::kan*); SA167 (RN4220 *ΔdivIVA*); SA213 (RN4220 *dnaK::kan ΔdivIVA*); SA266 (RN4220 *smc::Tn917*); SA269 (RN4220 *smc::Tn917ΔdivIVA*).*

We found no significant difference in growth rate or cell size in the Δ*divIVA dnaK::kan* double mutant (SA213) compared to the *dnakK::kan* mutant (SA210) in rich broth (BHI; **Table 1**) or minimal (chemically defined; CDM) media at 37°C (data not shown) indicating that any observable phenotype of the Δ*divIVA dnaK::kan* double mutant is not due to changes in growth or a delay in cell division in comparison to the *dnaK::kan* single mutant. Interestingly, a high proportion of the double mutant cells were anucleate when stained with DAPI and observed under the microscope (**Figure 3B**). This was similar in both rich and nutrient limiting media: 21% in BHI and 29% in CDM. Importantly, a higher frequency of anucleate cells were observed for the Δ*divIVA dnaK::kan* double mutant compared to *dnaK::kan* alone (two-fold higher; 21% vs. 10%, respectively). Further, a 10-fold and 1000-fold reduction in viability for the Δ*divIVA dnaK::kan* double mutant was also observed when stationary phase cells were plated on rich or nutrient-limiting solid media, respectively, compared to the *dnaK::kan* strain (**Figure 3C**). This reduction in viability was also observed in mid-exponential phase cultures grown in CDM media but not when cells were grown in BHI (Supplementary Figure 5) suggesting this reduction in viability is more apparent at reduced growth rates. Taken together, the higher frequency of anucleate cells, which will result in non-viable cells and a reduction in cell viability of the Δ*divIVA dnaK::kan* double mutant compared to the *dnaK* single mutant, supports the notion that DivIVA functions with DnaK in maintaining accurate chromosome segregation.

### Stability of DivIVA Requires DnaK

Since DnaK has a well characterized role as a molecular chaperone (Bukau and Horwich, 1998), the direct interaction we observed between DnaK and DivIVA may suggest a role for DnaK in maintaining DivIVA stability. To test this idea, we determined the cellular levels of DivIVA, as well as FtsZ and EzrA (which also showed an association with DnaK; **Figure 1**) in a *dnaK::kan* insertion mutant of *S. aureus* using western blot analysis. Cellular levels of both FtsZ and EzrA-GFP were similar in wild-type *S. aureus* (LH607 [*spa^-^*] for detection of FtsZ; SA353 [*spa^-^ezrA::ezrA-gfp* pGL485] for detection of EzrA-GFP) and the isogenic *dnaK::kan* mutant (SA359 [*spa^-^ dnaK::kan*] for detection of FtsZ; SA361 [*spa^-^dnaK::kan ezrA::ezrA-gfp* pGL485] for detection of EzrA-GFP; data not shown), demonstrating that DnaK is not required for the stability or septal localization of EzrA or FtsZ in *S. aureus* at 37°C.

**FIGURE 4:**
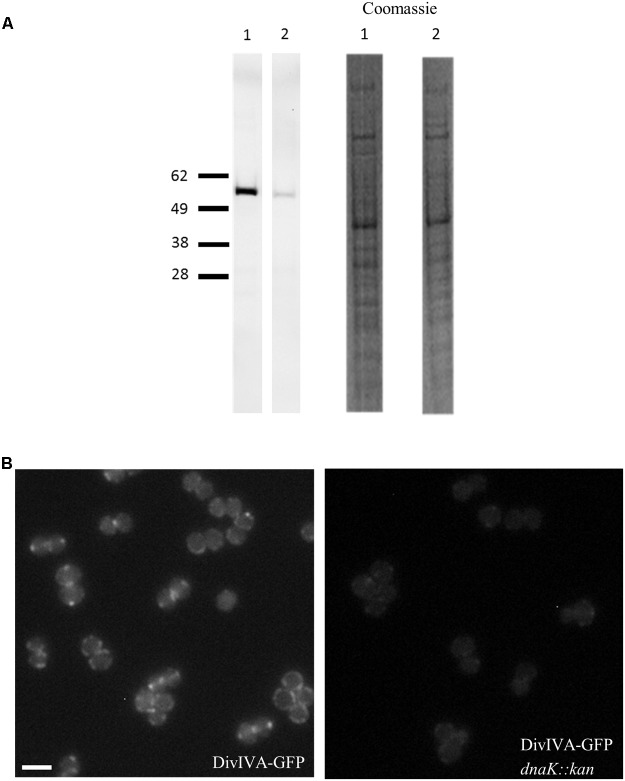
Reduction in DivIVA levels in the absence of *dnaK* in *S. aureus*. Immuno-detection of DivIVA as a GFP fusion protein in *S. aureus* cells expressing *divIVA-gfp* (SA356 [*spa^-^* P*_divIV A_ divIVA-gfp*::P*_spac_ divIVA*, pGL485]; **A**, Lane 1) and the *S. aureus dnaK::kan* mutant expressing *divIVA-gfp* (SA363 [*spa^-^ dnaK::kan* P*_divIV A_ divIVA-gfp*::P*_spac_ divIVA*, pGL485]; **A**, Lane 2) with anti-GFP antibodies. Densitometry analysis shows levels of DivIVA-GFP were reduced by 30 – 50% in SA356 compared to SA363. Coomassie stain of whole cell lysates are shown as a loading control. Fluorescence microscopy of *S. aureus* cells expressing (**B**, i) *divIVA-gfp* (SA247) and (**B**, ii) its isogenic *dnaK::kan* mutant (SA305) shows a reduction in the DivIVA-GFP signal. For microscopy, strains were grown in BHI broth at 37°C to mid-exponential phase of growth and visualized on 2% agarose pads, and identical exposure times were used to investigate the fluorescence intensity of DivIVA-GFP in the presence and absence of DnaK. For immuno-blotting, cell lysates were prepared from *S. aureus* strains grown to mid-exponential phase in BHI broth at 37°C. Scale bar is 2 μm.

Unlike EzrA and FtsZ, quantitative immunoblotting showed that cellular levels of DivIVA-GFP in the *S. aureus dnaK::kan* mutant (*spa^-^ dnaK::kan* P*_divIV A_ divIVA-gfp*::P*_spac_ divIVA* pGL485; SA363) were reduced by 30 – 50% compared to its parent *S. aureus* strain expressing *divIVA-gfp* (*spa^-^* P*_divIV A_ divIVA-gfp*::P*_spac_ divIVA* pGL485; SA356) (**Figure 4A**). Consistent with this result, fluorescence microscopy showed a marked decrease in the DivIVA-GFP signal intensity in the absence of *dnaK* (**Figure 4B**). It is important to note that the reduction in the levels of DivIVA-GFP in the absence of *dnaK* is unlikely to be caused by the presence of the GFP tag as EzrA-GFP levels remain unaffected by the loss of *dnaK* (data not shown). Therefore, these results strongly suggest that DnaK plays a specific role in maintaining the stability of DivIVA in *S. aureus*.

The anucleate phenotype of the *dnaK::kan* mutant of *S. aureus* cannot be solely due to the instability of DivIVA since the complete absence of *divIVA* does not yield a discernible phenotype. The *dnaK::kan* mutant phenotype is therefore likely to be additionally due to the instability of one of more additional proteins that depend on DnaK for their full stability.

DivIVA is highly conserved in Gram-positive bacteria (Oliva et al., 2010) and DnaK homologs are present in all bacteria (Bukau and Horwich, 1998). We therefore examined whether the requirement for DnaK in maintaining cellular DivIVA levels is conserved in *B. subtilis*. Indeed, DivIVA-GFP levels in the *B. subtilis dnaK::cat* mutant (*P_divIV A_ divIVA-gfp:: P_divIV A_ divIVA dnaK::cat*; SU798) were reduced by 35 – 50% when compared to the *B. subtilis* strain expressing *divIVA-gfp* (*P_divIV A_ divIVA-gfp:: P_divIV A_ divIVA*;SU761) (Supplementary Figures 6A,C). This reduction was also observed with untagged DivIVA (Supplementary Figure 6B). Together, these results demonstrate a requirement for DnaK in maintaining DivIVA stability in both organisms at 37°C.

### DivIVA Plays a Role in Chromosome Segregation

A role for DivIVA in chromosome segregation has been described previously in some bacterial species. For example, a *divIVA*::*kan* insertion mutant in *E. faecalis* resulted in an approximate100-fold decrease in viability and the majority of cells displayed anucleate cell phenotypes (Ramirez-Arcos et al., 2005) Previous studies in *B. subtilis* demonstrated that DivIVA functions in chromosome partitioning during sporulation by acting as a tether for RacA to anchor the chromosome origin region at the cell poles (Ben-Yehuda et al., 2003). Since our mutant data points to a role for *S. aureus* DivIVA in chromosome segregation, we examined whether DivIVA functions with known segregation proteins in *S. aureus*. As a first step to investigate this, we performed a bacterial two-hybrid analysis to determine if DivIVA interacts with six known *S. aureus* chromosome segregation proteins: SpoIIIE, FtsK, SMC, SpoOJ, ParC, ParE and the nucleoid occlusion protein, Noc, which displays amino acid sequence similarity to SpoOJ. Interestingly, we found a previously unknown interaction between *S. aureus* DivIVA and SMC (Supplementary Figure 7). No other pair-wise interactions with DivIVA were observed.

**FIGURE 5:**
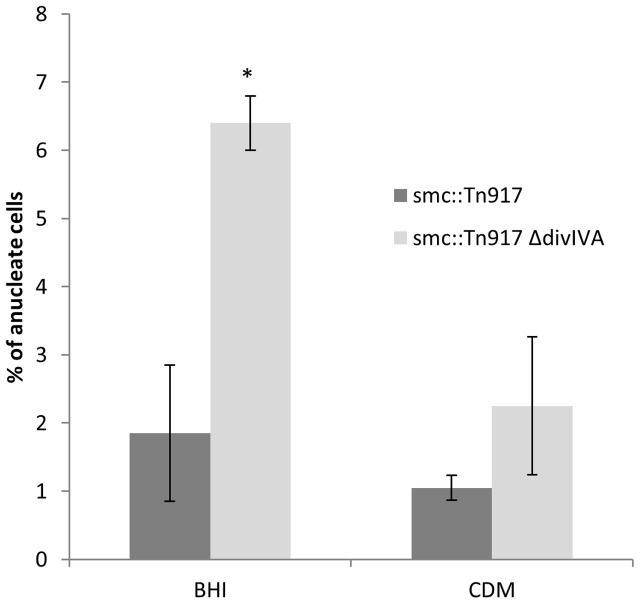
Increased frequency of anucleate cells in the double *smc::Tn917* Δ*divIVA* mutant compared to the *smc::Tn917* single mutant. Average percentages of anucleate cells in the *smc::Tn917* single mutant (SA266) and the *smc::Tn917* Δ*divIVA* double mutant (SA269). Both strains were grown in BHI media or CDM media at 37°C to mid-exponential phase of growth, stained with DAPI and visualized on 2% agarose pads. Anucleate cells were scored based on the absence of DAPI staining. Error bars (95% CIs) and averages were calculated from *n* = 300 cells from two independent experiments. The asterisk shows that, using the Fisher’s exact test, the number of anucleate cells is significantly higher in the double mutant (SA269) compared to the single mutant (SA266) when grown in BHI broth (*P*-value < 0.05).

The loss of *smc* in *S. aureus* is not lethal but results in the generation of anucleate cells at a frequency of 7% in the SA113 (ATCC 35556) background (Yu et al., 2010). If a *smc divIVA* double mutant shows an increase in the number of anucleate cells compared to the *smc* single mutant, it would support our proposal for the involvement of *divIVA* in chromosome segregation. Accordingly, the *divIVA smc* double mutant was constructed by introducing the *smc::Tn917* mutation (Yu et al., 2010) into RN4220 *ΔdivIVA* (SA167) creating the *smc::Tn917 ΔdivIVA* double mutant (SA269). In rich broth, the number of anucleate cells in the double mutant was about three-fold higher (Fisher’s exact test *p*-value < 0.05) than the *smc::Tn917* single mutant (6% ± 2% compared to 2% ± 1%) (**Figure 5**), suggesting that DivIVA and SMC act together to maintain accurate chromosome segregation.

## Discussion

In this study, we sought to identify novel divisome-associated components in the human pathogen *S. aureus* to better understand the spatial regulation of cell division in a spherical cell. We identified that the chaperone protein DnaK associated with divisome proteins FtsZ and EzrA, and also with DivIVA. Unlike FtsZ and EzrA, DivIVA is dependent on DnaK for stability, with DivIVA levels reduced to less than half in the absence of DnaK, thus identifying a novel substrate for the DnaK chaperone. We further observed that DivIVA levels were decreased in another Firmicute, *B. subtilis*, in the absence of DnaK. In other work, DnaK was co-isolated in complex with DivIVA in *B. subtilis* using a His-tag based pull-down technique (Halbedel et al., 2014), with DivIVA localization requiring the chaperone protein, SecA (Halbedel et al., 2014). These combined findings indicate that the correct folding of DivIVA, and therefore its localization, require chaperones. Interestingly, we found that DivIVA levels are not completely depleted in the absence of DnaK, suggesting that other chaperone systems such as SecA could also function to stabilize DivIVA in *S. aureus*. Our data provides evidence that the well-known chaperone DnaK has a function for maintaining the stability of DivIVA in *S. aureus*.

In the absence of DnaK, *S. aureus* forms anucleate cells and cells with enlarged diameters. A similar phenotype is also seen in *dnaK*-deleted *E. coli*, where anucleate and filamentous cells have been reported (Bukau and Walker, 1989; Bukau and Horwich, 1998; Sugimoto et al., 2008). This suggests an important chaperone function for DnaK in cell cycle processes such as cell division, resulting in enlarged cells, or chromosome segregation, resulting in anucleate cells. The observed increase in *S. aureus* cell size was not due to FtsZ or EzrA instability since in the absence of *dnaK* both are stable and localized correctly. However, in the absence of DnaK, DivIVA levels are reduced, resulting in an increase in anucleate cells. This indicates that there is a relationship between DnaK and the stability of DivIVA, which is important for the coordination between these cell cycle processes. It should be noted that there is likely redundancy in the regulatory processes coordinating chromosome segregation and cell division: since there is no observable phenotype in the absence of *divIVA*, the stability of DivIVA in the absence of *dnaK* does not fully explain the anucleate cells observed, suggesting there is another factor(s) that is involved in causing anucleate cells in the absence of *dnaK*. Nonetheless, we have shown in this study that DivIVA does contribute to the correct segregation of chromosomes in *S. aureus* – whether directly or via another factor – indicating it plays a role in coordinating chromosome segregation and cell division.

What is the function of DivIVA in *S. aureus*? Consistent with previous studies (Pinho and Errington, 2004), we found that the *S. aureus ΔdivIVA* mutant in both the RN4220 and SH1000 backgrounds showed no obvious phenotypic change when grown in both rich and defined media and a range of temperatures. However, when the *divIVA* deletion is combined with either a *dnaK* or *smc* deletion, we observed a significant increase in anucleate cells, indicating a role for DivIVA in coordinating chromosome segregation. Additionally, *S. aureus* DivIVA appears to only interact with SMC and no other chromosome segregation proteins. Our attempts to co-localize these two proteins was not possible. SMC was expressed as an RFP fusion both chromosomally under its native promoter or under an inducible promoter from a plasmid (Brzoska and Firth, 2013) but the fusion protein was non-functional, (data not shown). Future work investigating the localization, dynamics and co-dependency of SMC and DivIVA will therefore provide further insight into the role of DivIVA in co-ordinating cell cycle processes in this organism.

**FIGURE 6:**
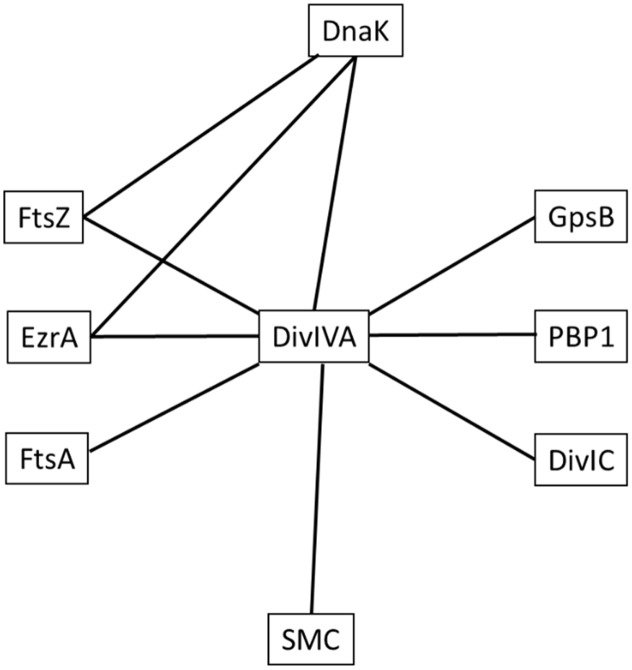
Network of interactions between *S. aureus* DivIVA, DnaK and divisome components. Lines indicate an observed interaction between proteins using the bacterial two-hybrid assay. The test proteins used in this study were DnaK and DivIVA and not every permutation of pairwise interactions was tested (see Supplementary Data for interactions tested in this study). Note that this interaction map is not exhaustive and only shows positive interactions that were detected in this study.

In *B. subtilis*, DivIVA has two primary roles. Firstly, during vegetative growth, it acts as a topological marker for the localization of the MinJCD protein complex to active septa to prevent Z-ring formation at newly formed cell poles (Bramkamp and van Baarle, 2009). A second role for *B. subtilis* DivIVA is the segregation of replicated chromosomes to each cell pole during spore development via interactions with the chromosome binding protein, RacA (Ben-Yehuda et al., 2003) and a complex containing ComN, MinJ, MinD and Soj (Kloosterman et al., 2016). Interestingly, as with *S. aureus*, *E. faecalis* has no Min system or a RacA homolog, but a *divIVA* deletion results in defects in both cell division and chromosome segregation (Ramirez-Arcos et al., 2005). Additionally, in actinobacteria which also lack a Min system, there is evidence that DivIVA is important for chromosome segregation: polar and septal localization of the centromere-binding protein ParB of *Corynebacterium glutamicum* requires DivIVA (Donovan et al., 2010), whilst *parB* mutants that can no longer interact with DivIVA result in a chromosome segregation defect (Donovan et al., 2012). Interactions between Wag31, the DivIVA homolog of *Mycobacterium tuberculosis*, and ParB, as well as DivIVA and ParB of *Streptomyces coelicolor* have also been observed (Donovan et al., 2012). Furthermore, there is evidence that DivIVA provides a link between chromosome segregation and cell division in bacteria which lack a Min system: interactions between DivIVA and both divisome and chromosome segregation proteins have been previously described in *S. pneumoniae* (Fadda et al., 2007) and actinobacteria (Letek et al., 2008; Sieger and Bramkamp, 2015). In contrast, *B. subtilis* DivIVA, that is part of the Min system of proteins, shows no direct interactions with components of septum formation machinery (Claessen et al., 2008), suggesting that the function of DivIVA has diverged, with the protein playing a role in spatial regulation of protein machineries in diverse species. In *S. aureus*, the interaction profile of DivIVA (**Figure 6**) and the observed phenotype of a *divIVA* mutant in combination with a known chromosome partitioning protein presented here indicates that DivIVA has a role in ensuring accurate co-ordination between chromosome segregation and the cell division processes.

As well as DivIVA localizing to the division septum in *S. aureus*, we could detect DivIVA-GFP at the cell periphery in a significant number of cells in the population (60% of cells showed both midcell and peripheral DivIVA localization and 2% showed DivIVA exclusively at the cell periphery). This has not been reported previously, and raises the possibility that, just prior to cell division, DivIVA is located peripherally in the curved membrane of the *S. aureus* cells where it can interact directly with SMC. It has been previously speculated that an *oriC*-binding protein could localize to the cell wall scars that are present in perpendicular planes of the previously two divisions in *S. aureus*, perhaps through interaction with member(s) of the divisome (Turner et al., 2010), with the protein becoming most highly concentrated at ‘poles’ where scars intersect, thus determining the division plane and directing chromosome segregation (Veiga et al., 2011). SMC is recruited to specific sites within the *oriC*-proximal third of the chromosome through direct interaction with Spo0J in several bacteria, including *B. subtilis* (Fadda et al., 2007; Gruber and Errington, 2009; Sullivan et al., 2009; Minnen et al., 2011; Donovan et al., 2012) where, in conjunction with Spo0J, it functions as a type of centromere. Although speculative, the observed additional non-septal localization of DivIVA and its interaction with SMC in *S. aureus* could reflect a function for DivIVA as an anchor at the cell ‘poles’ to facilitate chromosome segregation, with SMC being pulled away from midcell to clear the septal site of DNA for division to occur. A direct interaction between DivIVA and Spo0J has been observed in *S. pneumoniae* (Fadda et al., 2007), and DivIVA is known to anchor the *oriC* region during *B. subtilis* sporulation (Ben-Yehuda et al., 2003), raising the interesting question of how DivIVA coordinates chromosome segregation with cell division in different species. However, it cannot be ruled out that a currently unidentified DivIVA-interacting factor may be involved in coordinating chromosome segregation with cell division. While further studies are required to test this, our observation that a *divIVA smc* double mutant results in an increased frequency of anucleate cells, and detection of an interaction between these two proteins, suggests that SMC and DivIVA cooperate to ensure accurate chromosome segregation in *S. aureus*. Overall, the DivIVA localization pattern to both septal and peripheral sites, direct interactions with *S. aureus* divisome components and SMC suggests that DivIVA is a novel link between the cell division and chromosome segregation machinery.

## Materials and Methods

### Bacterial Strains, Plasmids and Oligonucleotides and Growth Conditions

*Bacillus subtilis* and *Staphylococcus aureus* strains used in this study are listed in Supplementary Table 1, plasmids are listed in Supplementary Table 2 and oligonucleotide sequences used (purchased from IDT) are shown in Supplementary Table 3.

### Growth Conditions and Media

*Escherichia coli* strains were grown in Luria-Bertani broth (LB; Oxoid). *S. aureus* strains were grown in brain heart infusion broth (BHI; Oxoid) or CDM (chemically defined media) as described previously. *B. subtilis* strains were grown in PAB medium (Difco Antibiotic Medium 3; BD). Unless otherwise stated, all strains were grown at 37°C. When required, antibiotics were added to a final concentration of 100 μg ml^-1^ ampicillin, 10 μg ml^-1^ tetracycline, 5 μg ml^-1^ erythromycin, 25 μg ml^-1^ lincomycin, 50 μg ml^-1^ kanamycin, 10 μg ml^-1^ spectinomycin, 10 μg ml^-1^ chloramphenicol (5 μg ml^-1^ for selection in *B. subtilis*).

Growth rate experiments were performed in triplicate in 96-well flat bottom plates. 150 μl fresh medium (supplemented with 0.05 M IPTG when necessary) was inoculated to an OD_600_ of 0.02 and growth was monitored every 30 min with moderate shaking at the required temperature using a PowerWave HT Microplate Spectrophotometer (Bio-Tek). An unpaired student *t*-test was used to compare the generation time of RN4220, SA210 (*dnaK::kan*) and SA213 (*ΔdivIVA dnaK::kan*).

Gross viability assays were performed as follows: mid-exponential or stationary phase cultures were normalized to an OD_600_ value of 1, serially diluted (10^-3^ to 10^-7^) in PBS and 10 μl was drop-plated onto BHI or CDM agar. Plates were then air dried and incubated for 24 h at 37°C.

Transformations of *S. aureus* RN4220 were performed using electroporation as described previously (Kraemer and Iandolo, 1990). Phage transductions into non-RN4220 *S. aureus* backgrounds using φ11 were performed as described previously (Novick, 1967). *B. subtilis* SU5 *dnaK::cat* mutants (SU797 and SU798) were constructed by transferring the *dnaK::cat* construct from strain BT02 (Schulz et al., 1995) into the SU5 background via natural transformation and homologous recombination using purified genomic DNA from BT02. DNA manipulations and *E. coli* transformations were performed using standard molecular biology techniques.

### Microscopy Methods

Samples were prepared for live cell microscopy as previously described (Pereira et al., 2010) and visualized on a 2% agarose pad. Cells were observed using a Zeiss Axioplan 2 fluorescence microscope equipped with a Plan ApoChromat (100x NA 1.4; Zeiss) objective lens and an AxioCam MRm cooled charged-coupled-device (CCD) camera. To visualize DNA and cell membranes, 1 μg ml^-1^ DAPI (Molecular Probes) and 0.25 μg ml^-1^ FM 4-64 (Life Technologies) were incubated with *S. aureus* for 5 and 30 min respectively.

Cell length values were measured directly from digital micrographs using AxioVision software, version 4.6 (Zeiss) with default measurement settings. The Fisher’s exact test was used to compare the number of anucleate cells in the *dnaK* mutants and was performed with Graphpad Prism software.

### Construction of *dnaK* Containing Plasmids pLOW and pLOW-msfGFP

To construct plasmid pLOW-*dnaK*, primers 1280 and 1281, containing *Sal*I and *Bam*HI restriction sites respectively, were used to PCR amplify full-length *dnaK* from *S. aureus* RN4220 gDNA and then cloned into plasmid pLOW.

pLOW-*msf*GFP was constructed by amplification of msfGFP from pDHL1029 (Landgraf et al., 2012) using primer pair 1161 and 1162, and ligation into pLOW using complementary *Xma*I and *Eco*RI sites. pLOW-*dnaK-msfGFP* was subsequently constructed as for pLOW-*dnaK*.

### Construction of Fluorescent Derivatives of *S. aureus* DivIVA and DnaK

Plasmid pBCB1-GE IVA700 was used to construct strain SA247 (RN4220 P*_divIV A_ divIVA-gfp*::P*_spac_ divIVA*, pGL485) which expresses *divIVA-gfp* from its native promoter while placing full length *divIVA* under the control of the P*_spac_* promoter. Full length *S. aureus divIVA* was PCR amplified from RN4220 gDNA using primers 1438 and 1452 containing flanking *Kpn*I restriction sites. The PCR product was digested and inserted into pBCB1-GE, to create plasmid pBCB1-GE IVA700. The orientation and sequence of the cloned insert was verified before transformation into RN4220.

pBCB1-GE P*_divIV A_ divIVA-gfp* was used to construct strain SA289 (RN4220 pBCB1-GE *P_divIV A_ divIVA-gfp*) which expresses both *divIVA-gfp* and full length *divIVA* from the native *divIVA* promoter. First, full length *S. aureus divIVA* and the 5’ 200 bps region upstream of *divIVA* (containing its native promoter) was PCR amplified from RN4220 gDNA using primers 1471 and 1472 containing *Kpn*I restriction sites. The PCR product, along with plasmid pBCB1-GE, was digested with *Kpn*I and ligated, creating plasmid pBCB1-GE P*_divIV A_ divIVA-gfp*. The orientation and sequence of the cloned insert was verified before transformation into RN4220.

pBCB1-GE 3’*dnaK* was used to construct strain SA220 (RN4220 *dnaK::dnaK-gfp*, pGL485) which expresses a *dnaK* C-terminal GFP fusion from the native *dnaK* promoter. To do this, a 3′ 800 bps *dnaK* fragment was PCR amplified from RN4220 gDNA with primer pair 1314 and 1315 (containing *Kpn*I restriction sites) and cloned in-frame with *gfp* downstream of the P*_spac_* promoter on plasmid pBCB1-GE. The orientation and sequence of the cloned insert was verified and transferred to RN4220.

### Construction of an *S. aureus divIVA* Deletion Strain

The thermosensitive plasmid pMAD (Arnaud et al., 2004) was used to make a *divIVA* null mutant in the laboratory strain SH1000 background (LC102; SH1000 *spa::tet*). Briefly, PCR fragments containing ∼1.5 kb flanking regions of *divIVA* were amplified from SH1000 gDNA using primers 1565/1566 and 1567/1568. The PCR products were fused together by Gibson Assembly following manufacturer’s instructions (NEB), followed by gel extraction of the DNA fragment of correct size. This DNA fragment was digested with *Bam*HI and *Bgl*II and cloned into pMAD. The plasmid was electroporated into RN4220 at 30°C and subsequently transduced into LC102 at 30°C. Integration of the plasmid into the chromosome was achieved by inoculating BHI with single colonies and incubating at 42°C for 6 h before spreading on BHI agar in the absence of antibiotics and incubation overnight at 42°C. To then allow double cross-over events to occur, resulting in the deletion of *divIVA*, colonies were initially grown in BHI medium at 37°C overnight, and then subcultured at 25°C for two 24 h passages. The culture was plated in the absence of antibiotics and incubated at 37°C (Kato and Sugai, 2011). Colonies that showed erythromycin sensitivity and were not blue on X-gal were screened by PCR using primers 1563/1564 to confirm the deletion of *divIVA* to create strain SA365 (SH1000 *spa^-^ ΔdivIVA*). No difference in growth rate, viability, cell size or anucleate cells were observed in SA365 compared to LC102. This is in agreement with results seen for RN4220Δ*divIVA* (this study; Pinho and Errington, 2004).

### Bacterial Two-Hybrid Analyses

To screen for interactions of DnaK and DivIVA with various proteins involved in cell division or chromosome segregation, the coding sequences of *dnaK, divIVA*, *spoIIIE*, *ftsK*, *smc* and *spoOJ* were amplified by PCR using appropriate primer pairs (Supplementary Table 3) and cloned into pUT18 (Karimova et al., 1998) and p25-N (Claessen et al., 2008) using restriction sites *Bam*HI and *Sac*I (*spoIIIE, ftsK, spoOJ, dnaK*), *Bam*HI and *Kpn*I (*smc*) or *Bam*HI and *Eco*RI (*divIVA*) to create C-terminal fusions of the proteins to the T18 or T25 domain of adenylate cyclase respectively. N-terminal fusions of DnaK were also created by PCR amplifying the *dnaK orf* using primer pairs 1285/1286 and 1289/1290 which introduces flanking *Bam*HI-*Sac*I and *Bam*HI-*Kpn*I sites, respectively, to the PCR products. Inserts were cloned into plasmid pUT18C and pKT25 (Karimova et al., 1998) using the same sites.

To test for pair-wise protein-protein interactions in the BACTH assay, plasmids were co-transformed into BTH101 and β-galactosidase activity produced from the *E. coli* strains was qualitatively assessed by observing the blue coloration of the cleaved X-Gal substrate as described previously (Steele et al., 2011). Plates are representative from at two independent experiments.

### Quantitative Immunoblotting

*Staphylococcus aureus* LH607 (NCTC 8325-4 *spa::*tet) was used for the immunoblots to prevent non-specific binding of antibodies to Protein A (Pinho and Errington, 2003). Strains were grown to mid-exponential phase and normalized to an OD_600_ value of 1 before collection of cells (∼5 ml) by centrifugation and resuspension in WL buffer ([0.3 mg ml^-1^ lysostaphin for *S. aureus*; 0.3 mg ml^-1^ lysozyme for *B. subtilis*], 1x protease inhibitor (Roche), 25 mM Tris, 0.3 mg ml^-1^ PMSF). Cell lysis was performed at 37°C for 30 min and protein concentration was determined by Bradford assay. Samples were separated on 4–12% NuPAGE Novex bis-Tris electrophoresis gels (Invitrogen) and transferred to a PVDF membrane by either 7-min semi-dry western transfer method (iBlot, Invitrogen) or standard wet transfer. Blots were probed with *B. subtilis* FtsZ antiserum (a kind gift from Moriya S.), anti-GFP antibodies (Roche) or *B. subtilis* DivIVA antiserum (Marston et al., 1998) at 1:10000, 1:1000 or 1:5000 dilutions, respectively. Immunodetection was performed using the ECL kit (GE) according to the manufacturer’s recommendations. Densitometry analysis was performed using the Quantity One software (Bio-Rad). Percentages indicate the densitometry values of the mutant when compared to its isogenic parent strain.

### Protein Complex Isolation

#### Cryogenic Lysis of *S. aureus* Cells for Protein Complex Isolation

*Staphylococcus aureus* LH607 (8325-4 *spa*^-^) cells expressing *ftsZ-gfp* (SA103) or untagged-*gfp* (SA112) were grown to mid-exponential phase in 4 L of BHI broth at 37°C in the presence of 0.05 mM IPTG and harvested by centrifugation at 4000 × *g* for 15 min. The resulting pellet was washed three times with 5 ml of 0.1 M NaH_2_PO_4_ and then resuspended in 2 ml of 0.1 M NaH_2_PO_4_. Cell biomass was normalized between samples using OD_600_ readings before cross-linking protein complexes with a final concentration of 1% (w/v) formaldehyde for 20 min at 37°C with mixing at 5-min intervals. To quench the cross-linking reaction, 1 ml of 2 M Tris/1.2 M glycine was added and incubated at 37°C for 20 min with mixing at 1-min intervals. Cells were collected by centrifugation and washed three times with PBS. The cell pellet was then snap-frozen in liquid nitrogen and vacuum freeze dried overnight to improve the efficiency of the cell lysis. Freeze-dried cell pellets were subjected to cryogenic grinding using a Mikro-Dismembrator II (Braun, Germany) inside pre-chilled 5 ml stainless steel shaker flasks containing two 7 mm stainless steel grinding balls at maximum amplitude. The jars were cooled with liquid nitrogen between each grinding cycle. The efficiency of lysis was determined by phase-contrast microscopy.

#### Conjugation of M-270 Epoxy Dynabeads with Polyclonal Anti-GFP Antibody

Conjugation of dynabeads to anti-GFP antibody was performed as previously described (Cristea et al., 2005; Cristea and Chait, 2011) using 10 mg of M-270 Epoxy magnetic Dynabeads (Invitrogen) and 50 μg of anti-GFP antibody. Conjugated beads were stored in 500 μl of PBS containing 0.02% NaN_3_ at 4°C.

### FtsZ-GFP Protein Complex Purification in *S. aureus*

Protein complexes were isolated from cryogenically lysed cells following the method of Cristea et al. (2005) using 5 mg anti-GFP conjugated M-270 Epoxy Dynabeads. The eluted fraction snap frozen in liquid nitrogen and dried in a vacuum concentrator (Eppendorf) for 4.5 h. The resulting pellet was resuspended in SDS-PAGE sample buffer and separated on a 4–12% NuPAGE Novex bis-Tris electrophoresis gel (Invitrogen) prior to mass spectrometry.

### Identification of Proteins by Mass Spectrometry

Sample lanes were excised from the gel and cut into smaller gel pieces which were then reduced, alkylated and digested overnight with trypsin. Digested peptides were separated by nano-LC using an Ultimate 3000 HPLC and autosampler system (Dionex). Samples (2.5 μl) were concentrated and desalted onto a micro C18 precolumn (500 μm × 2 mm, Michrom Bioresources, Auburn, CA, United States) with H_2_O:CH_3_CN (98:2, 0.05% HFBA) at 20 μl/min. After a 4 min wash the pre-column was switched into line with a fritless nano column (75 μ ×∼10 cm) containing C18 media (5 μ, 200 Å Magic, Michrom) manufactured according to specifications described previously (Gatlin et al., 1998). Peptides were eluted using a linear gradient of H_2_O:CH_3_CN [98:2, 0.1% (v/v) formic acid] to H_2_O:CH_3_CN [64:36, 0.1% (v/v) formic acid] at 350 nl/min over 30 min. A high voltage of 1800 V was applied to low volume tee (Upchurch Scientific) and the column tip positioned ∼0.5 cm from the heated capillary (T = 250°C) of a LTQ FT Ultra (Thermo Electron, Bremen, Germany) mass spectrometer. Positive ions were generated by electrospray and the LTQ FT Ultra operated in data dependent-acquisition mode (DDA).

A survey scan m/z 350-1750 was acquired in the FT ICR cell (Resolution = 100,000 at m/z 400, with an initial accumulation target value of 1,000,000 ions in the linear ion trap). Up to the 6 most abundant ions (>3,000 counts) with charge states of +2, +3, or +4 were sequentially isolated and fragmented within the linear ion trap using collisionally induced dissociation with an activation *q* = 0.25 and activation time of 30 ms at a target value of 30,000 ions. M/z ratios selected for MS/ MS were dynamically excluded for 30 s. Peak lists were generated using Mascot Daemon/extract_msn (Matrix Science, London, England, Thermo) using the default parameters, and submitted to the database search program Mascot (version 2.1, Matrix Science). Search parameters were: Precursor tolerance 4 ppm and product ion tolerances ± 0.4 Da; Met(O) specified as variable modification, enzyme specificity was trypsin, 1 missed cleavage was possible and the non-redundant IPI_human protein database (May 2009) searched.

Proteins that are identified as “significant hits” contain at least 2 peptides with a Mascot score of >100 from 2 independent experiments (Cooper et al., 2009). Proteins that were present in both the FtsZ-GFP sample and the GFP control sample were considered to be non-specific interacting proteins and were excluded from further analysis.

## Author Contributions

AB, AL, KK, EP, and EH designed experiments; AB, AL, KK, EP, and LS performed experiments; AB, AL, SF, and EH analyzed data; AB, AL, SF, and EH prepared the manuscript. All authors read and edited the manuscript.

## Conflict of Interest Statement

The authors declare that the research was conducted in the absence of any commercial or financial relationships that could be construed as a potential conflict of interest.
