# Supplementary material for: Coordination of Chromosome Segregation and Cell Division in Staphylococcus aureus
Source: Front Microbiol. 2017 Aug 23;8:1575. doi: 10.3389/fmicb.2017.01575 (PMC5572376; doi:10.3389/fmicb.2017.01575)
Supplement: Supplementary file 1 [file Data_Sheet_1.DOCX]

Supplementary Table 1 *S. aureus* and *B. subtilis* strains

| Strain | Description^a^ | Construction | Reference |
| --- | --- | --- | --- |
| *S. aureus* | | |  |
| jGL227 | SH1000 *ezrA::ezrA-gfp*, pGL485 (*erm^R^ cat^R^*) |  | (Steele et al. 2011) |
| LH607 | 8325-4 *spa*::*tet* (*tet^R^*) |  | (Pinho & Errington 2003) |
| LC102 | SH1000 *spa*::*tet* (*tet^R^*) |  | (Cooper et al. 2009) |
| RN4220 | 8325-4 restriction deficient strain which accepts *E. coli*-extracted DNA but contains a mutation affecting SigmaB activity |  | (Kreiswirth et al. 1983) |
| SA94 | SH1000 pLOW *ftsZ-gfp*, pGL485 (*erm^R^ cat^R^*) |  | (Liew et al. 2011) |
| SA102 | LH607 pGL485 (*tet^R^ cat^R^*) |  | This study |
| SA103 | LH607 pLOW *ftsZ-gfp*, pGL485 (*erm^R^, cat^R^, tet^R^*) | SA94→SA102 | This study |
| SA111 | SH1000 pLOW GFP, pGL485 (*erm^R^, cat^R^, tet^R^*) |  | (Liew et al. 2011) |
| SA112 | LH607 pLOW GFP, pGL485 (*tet^R^, erm^R^ cat^R^*) | SA111→SA102 | This study |
| SA167 | RN4220 *∆divIVA* |  | (Pinho & Errington 2004) |
| SA182 | NCTC8325-4 *dnaK::kan* (*kan^R^*) |  | (Singh et al. 2007) |
| SA210 | RN4220 *dnaK:kan* (*kan^R^)* | SA182→RN4220 | This study |
| SA213 | RN4220 ∆*divIVA* *dnaK:kan* (*kan^R^)* | SA182→SA167 | This study |
| SA220 | RN4220 *dnaK::dnaK-gfp*, pGL485 (*erm^R^ cat^R^*) | pBCB1-GE 3’*dnaK*, pGL485→RN4220 | This study |
| SA247 | RN4220 P*_divIVA_ divIVA-gfp*::P*_spac_* *divIVA*, pGL485 (*erm^R^ cat^R^*) | pBCB1-GE IVA700, pGL485→RN4220 | This study |
| SA260 | SA113 *smc::Tn917* (*erm^R^)* |  | (Yu et al. 2010) |
| SA266 | RN4220 *smc::Tn917* (*erm^R^)* | SA260→RN4220 | This study |
| SA269 | RN4220 ∆*divIVA* *smc::Tn917* (*erm^R^)* | SA260→SA167 | This study |
| SA289 | RN4220 pBCB1-GE *P_divIVA_ divIVA-gfp* (*erm^R^)* | pBCB1-GE P*_divIVA_* *divIVA-gfp*→RN4220 | This study |
| SA303 | RN4220 *dnaK:kan* pLOW *dnaK*, pGL485 (*erm^R^ cat^R^*) | pLOW *dnaK*, pGL485→SA210 | This study |
| SA305 | RN4220 *dnaK::kan* P*_divIVA_ divIVA-gfp*::P*_spac_* *divIVA*, pGL485 *(kan^R^ erm^R^ cat^R^, tet^R^*) | SA182, pGL485→SA255 | This study |
| SA307 | RN4220 pLOW *dnaK-msgfp*, pGL485 (*erm^R^ cat^R^*) | pLOW *dnaK-msfgfp*, pGL485→RN4220 | This study |
| SA316 | RN4220 *ezrA::ezrA-gfp*, pGL485 (*erm^R^ cat^R^*) | jGL227→RN4220 | This study |
| SA320 | RN4220 *dnaK*::*kan* *ezrA::ezrA-gfp*, pGL485 (*kan^R^ erm^R^ cat^R^*) | SA182→SA316 | This study |
| SA353 | LH607 *ezrA::ezrA-gfp*, pGL485 (*erm^R^ cat^R^*) | jGL227→SA102 | This study |
| SA356 | LH607 P*_divIVA_ divIVA-gfp*::P*_spac_* *divIVA*, pGL485 *(erm^R^ cat^R^ tet^R^*) | SA247→SA102 | This study |
| SA359 | LH607 *dnaK::kan* (*kan^R^ tet^R^* ) | SA182→LH607 | This study |
| SA361 | LH607 *dnaK::kan*, *ezrA::ezrA-gfp*, pGL485(*kan^R^ erm^R^ cat^R^ tet^R^*) | SA182→SA353 | This study |
| SA363 | LH607 *dnaK::kan* P*_divIVA_ divIVA-gfp*::P*_spac_* *divIVA*, pGL485 *(kan^R^ erm^R^ cat^R^ tet^R^*) | SA182→SA356 | This study |
| SA365 | LC102 *∆divIVA* | pMAD_divIVA_KO→LC102 | This study |
| *B. subtilis* | | | |
| SU5 | 168 *trpC2* |  | Lab stock |
| BT02 | *B. subtilis* 1012 *dnaK::cat* (*cat^R^*) |  | (Schulz et al. 1995) |
| SU761 | *B. subtilis* SU5 *P_divIVA_ divIVA-gfp:: P_divIVA_ divIVA* (*tet^R^*) |  | Lab stock |
| SU797 | *B. subtilis* SU5 *dnaK::cat* (*cat^R^*) | BT02→SU5 | This study |
| SU798 | *B. subtilis* SU761 *dnaK::cat* (*tet^R^* *cat^R^*) | BT02→SU761 | This study |

^a^Antibiotic resistance markers are expressed as follows: *tet^R^*; tetracycline resistance, *erm^R^*; erythromycin resistance, *cat****^R^***; chloramphenicol resistance, *kan^R^*; kanamycin resistance. Arrow refers to: 1) electroporation of plasmids into RN4220, 2) phage transduction of a *S. aureus* construct from one strain into a different strain background or 3) the transfer of a *B. subtilis* construct from one strain into a different strain background via natural transformation using genomic DNA.

SupplementaryTable 2 Plasmids used in this study

| Plasmid | Description^a^ | Reference |
| --- | --- | --- |
| p25N | Low copy-number derivative of pSU40 encoding the T25 fragment, corresponding to the first 224 amino acids of CyaA, downstream of a multiple cloning site; Kan^r^ | (Claessen et al. 2008) |
| p25N-*dnaK* | p25N containing the 3’ end of *dnaK* fused in frame to T25; Kan^r^ | This study |
| pALB50 | p25N containing the 3’ end of *divIVA* fused in frame to T25; Kan^r^ | This study |
| pVF30 | p25N containing the 3’ end of *ezrA* fused in frame to T25; Kan^r^ | (Steele et al. 2011) |
| p25N-*ftsK* | p25N containing the 3’ end of *ftsK* fused in frame to T25; Kan^r^ | This study |
| pVF29 | p25N containing the 3’ end of *ftsZ* fused in frame to T25; Kan^r^ | (Steele et al. 2011) |
| p25N-*smc* | p25N containing the 3’ end of *smc* fused in frame to T25; Kan^r^ | This study |
| p25N-*spoIIIE* | p25N containing the 3’ end of *spoIIIE* fused in frame to T25; Kan^r^ | This study |
| p25N-*spoOJ* | p25N containing the 3’ end of *spoOJ* fused in frame to T25; Kan^r^ | This study |
| pBCB-1GE | *S. aureus* integrative vector for generating N- and C-terminal GFP fusions; Amp^r^, Erm^r^ | (Pereira et al. 2010) |
| pBCB1-GE 3’*dnaK* | Plasmid pBCB1-GE containing a 3’ 800 bps *dnaK* fragment cloned in-frame with *gfp* downstream of the P*spac* promoter; Amp^r^, Erm^r^ | This study |
| pBCB1-GE IVA700 | Plasmid pBCB1-GE containing full length *divIVA* cloned in-frame with *gfp* downstream of the P*spac* promoter; Amp^r^, Erm^r^ | This study |
| pBCB1-GE P*_divIVA_* *divIVA-gfp* | Plasmid pBCB1-GE containing native upstream promoter and full length *divIVA* cloned in-frame with *gfp* downstream of the P*spac* promoter; Amp^r^, Erm^r^ | This study |
| pDHL1029 | Plasmid containing a monomeric superfolder GFP (msfGFP) tag. The superfolder GFP *orf* contains the A206K mutation. Amp^r^ | (Landgraf et al. 2012) |
| pGL485 | High-copy number staphylococcal plasmid containing a constitutively expressed lacI repressor; Spec^r^, Cat^r^ | (Cooper et al. 2009) |
| pKT25 | Low copy-number derivative of pSU40 encoding the T25 fragment of *B. pertussis* adenylate cyclase, corresponding to the first 224 amino acids of CyaA, upstream of a multiple cloning site; Kan^r^ | (Karimova et al. 1998) |
| pGL540 | pKT25 containing the 5’ end of *divIB* fused in frame to T25; Kan^r^ | (Steele et al. 2011) |
| pGL551 | pKT25 containing the 5’ end of *divIC* fused in frame to T25; Kan^r^ | (Steele et al. 2011) |
| pKT25-*dnaK* | pKT25 containing the 5’ end of *dnaK* fused in frame to T25; Kan^r^ | This study |
| pGL541 | pKT25 containing the 5’ end of *ftsA* fused in frame to T25; Kan^r^ | (Steele et al. 2011) |
| pGL542 | pKT25 containing the 5’ end of *ftsL* fused in frame to T25; Kan^r^ | (Steele et al. 2011) |
| pALB3 | pKT25 containing the 5’ end of *ftsW* fused in frame to T25; Kan^r^ | (Steele et al. 2011) |
| pGL557 | pKT25 containing the 5’ end of *gpsB* fused in frame to T25; Kan^r^ | (Steele et al. 2011) |
| pGL560 | pKT25 containing the 5’ end of *yyaA* fused in frame to T25; Kan^r^ | J. Kasturiarachchi, unpublished |
| pGL553 | pKT25 containing the 5’ end of *parC* fused in frame to T25; Kan^r^ | J. Kasturiarachchi, unpublished |
| pGL554 | pKT25 containing the 5’ end of *parE* fused in frame to T25; Kan^r^ | J. Kasturiarachchi, unpublished |
| pGL543 | pKT25 containing the 5’ end of *pbp2* fused in frame to T25; Kan^r^ | (Steele et al. 2011) |
| pGL556 | pKT25 containing the 5’ end of *pbp3* fused in frame to T25; Kan^r^ | (Steele et al. 2011) |
| pGL550 | pKT25 containing the 5’ end of *pbpA* fused in frame to T25; Kan^r^ | (Steele et al. 2011) |
| pALB8 | pKT25 containing the 5’ end of *rodA* fused in frame to T25; Kan^r^ | (Steele et al. 2011) |
| pGL559 | pKT25 containing the 5’ end of *ylmf* fused in frame to T25; Kan^r^ | (Steele et al. 2011) |
| pKT25-zip | pKT25 containing the 5’ end of the leucine zipper of GCN4 fused in frame to T25 and serves as a positive control for β-galactosidase activity; Kan^r^ | (Karimova et al. 1998) |
| pLOW | Low-copy number staphylococcal shuttle vector containing the IPTG inducible P*spac* promoter and lacI repressor; Amp^r^ Erm^r^ | (Liew et al. 2011) |
| pLOW-*dnaK* | pLOW with full length *dnaK* expressed under the control of the P*spac* promoter; Amp^r^, Erm^r^ | This study |
| pLOW-*dnaK-msfGFP* | pLOW with *dnaK*, fused to superfolder GFP under the control of the P*spac* promoter; Amp^r^, Erm^r^ | This study |
| pLOW-GFP | pLOW with a GFP tag located downstream of the multi-cloning site for constructing GFP fusion proteins; Amp^r^, Erm^r^ | (Liew et al. 2011) |
| pLOW GFP-*ftsZ* | pLOW with *gfp* upstream of *ftsZ* expressed under the control of the P*spac* promoter; Amp^r^, Erm^r^ | (Liew et al. 2011) |
| pLOW-msfGFP | pLOW with superfolder GFP located downstream of the multi-cloning site for constructing monomeric GFP fusion proteins; Amp^r^, Erm^r^ | This study |
| pUT18 | High copy-number derivative of pUC19 encoding the T18 fragment, corresponding to amino acids 225 to 399 of CyaA, downstream of a multiple cloning site; Amp^r^ | (Karimova et al. 1998) |
| pALB46 | pUT18 containing the 3’ end of *divIVA* fused in frame to T18; Amp^r^ | This study |
| pUT18-*dnaK* | pUT18 containing the 3’ end of *dnaK* fused in frame to T18; Amp^r^ | This study |
| pVF32 | pUT18 containing the 3’ end of *ezrA* fused in frame to T18; Amp^r^ | (Steele et al. 2011) |
| pUT18-*ftsK* | pUT18 containing the 3’ end of *ftsK* fused in frame to T18; Amp^r^ | This study |
| pVF31 | pUT18 containing the 3’ end of *ftsZ* fused in frame to T18; Amp^r^ | (Steele et al. 2011) |
| pUT18-*smc* | pUT18 containing the 3’ end of *smc* fused in frame to T18; Amp^r^ | This study |
| pUT18-*spoIIIE* | pUT18 containing the 3’ end of *spoIIIE* fused in frame to T18; Amp^r^ | This study |
| pUT18-*spoOJ* | pUT18 containing the 3’ end of *spoOJ* fused in frame to T18; Amp^r^ | This study |
| pUT18C | High copy-number derivative of pUC19 encoding the T18 fragment, corresponding to amino acids 225 to 399 of CyaA, upstream of a multiple cloning site; Amp^r^ | (Karimova et al. 1998) |
| pGL544 | pUT18C containing the 5’ end of *divIB* fused in frame to T18; Amp^r^ | (Steele et al. 2011) |
| pGL564 | pUT18C containing the 5’ end of *divIC* fused in frame to T18; Amp^r^ | (Steele et al. 2011) |
| pUT18C-*dnaK* | pUT18C containing the 5’ end of *dnaK* fused in frame to T18; Amp^r^ | This study |
| pGL545 | pUT18C containing the 5’ end of *ftsA* fused in frame to T18; Amp^r^ | (Steele et al. 2011) |
| pGL546 | pUT18C containing the 5’ end of *ftsL* fused in frame to T18; Amp^r^ | (Steele et al. 2011) |
| pALB6 | pUT18C containing the 5’ end of *ftsW* fused in frame to T18; Amp^r^ | (Steele et al. 2011) |
| pGL570 | pUT18C containing the 5’ end of *gpsB* fused in frame to T18; Amp^r^ | (Steele et al. 2011) |
| pGL573 | pUT18C containing the 5’ end of *yyaA* fused in frame to T18; Amp^r^ | J. Kasturiarachchi, unpublished |
| pGL547 | pUT18C containing the 5’ end of *pbp2* fused in frame to T18; Amp^r^ | (Steele et al. 2011) |
| pALB14 | pUT18C containing the 5’ end of *rodA* fused in frame to T18; Amp^r^ | (Steele et al. 2011) |
| pGL572 | pUT18C containing the 5’ end of *ylmf* fused in frame to T18; Amp^r^ | (Steele et al. 2011) |
| pUT18C-zip | pUT18C containing the 5’ end of the leucine zipper of GCN4 fused in frame to T18 and serves as a positive control for β-galactosidase activity; Amp^r^ | (Karimova et al. 1998) |

^a^Antibiotic resistance markers are expressed as follows: Amp*^R^*; Ampicillin resistance, *tet^R^*; tetracycline resistance, *erm^R^*; erythromycin resistance, *cat*; chloramphenicol resistance, *kan^R^*; kanamycin resistance, P*_spac_*; IPTG-inducible promoter.

Supplementary Table 3 Primers synthesized in this study

| Primer | Sequence (5’to 3’)^a^ | F or R^b^ | Application/description |
| --- | --- | --- | --- |
| 1161 | ATTACCCGGGATGAGTAAAGGAGAAG | F | PCR primers containing *Xma*I and *EcoR*I restriction sites respectively, used to PCR amplify the *msfGFP* orf for cloning into plasmid pLOW for the construction of pLOW *msf*GFP. |
| 1162 | TCTAGAATTCGTGGATCTGAAGTCTG | R |  |
| 1280 | GATTGTCGACTAAATTAAATGGAGGAATTTTATTATGAG | F | PCR primer pairs containing *Sal*I and *BamH*I restriction sites respectively, used to PCR amplify the *dnaK orf* for cloning into plasmid pLOW for the construction of pLOW- *dnaK*. |
| 1281 | TTAAGGATCCGACAACATTCTAATTGTATTGTTTAATTA | R |  |
| 1283 | GATTGGATCCTATGAGTAAAATTATTGGTATAGAC | F | PCR primer pairs containing *BamH*I and *Sac*I restriction sites respectively, used to PCR amplify the *dnaK orf* for cloning into plasmid pUT18 and p25N for the construction of pUT18-*dnaK* and p25N-*dnaK*. |
| 1284 | TTAAGAGCTCAATTTTTTGTCGTCGTCTTTTACTTC | R |  |
| 1285 | GATTGTCGACTATGAGTAAAATTATTGGTATAGAC | F | PCR primer pairs containing *Sal*I and *BamH*I restriction sites respectively, used to PCR amplify the *dnaK orf* for cloning into plasmid pUT18C for construction of plasmid pUT18C-*dnaK*. The primers were also used to confirm disruption of the *dnaK orf* in *S. aureus dnaK* mutants. |
| 1286 | TTAAGGATCCTTATTTTTTGTCGTCGTCTTTTACTTC | R |  |
| 1289 | GATTGGATCCAATGAGTAAAATTATTGGTATAGAC | F | PCR primer pairs containing *BamH*I and *Kpn*I restriction sites, respectively, used to PCR amplify the *dnaK orf* for cloning into plasmid pKT25 for construction of plasmid pKT25-*dnaK*. |
| 1290 | TTAAGGTACCTTATTTTTTGTCGTCGTCTTTTACTTC | R |  |
| 1314 | ATAATAGGTACCACGTTAATTGAACGTAACACTACGATTC | F | PCR primer pairs containing flanking *Kpn*I restriction sites, used to PCR amplify a 500 bps fragment containing the 3’ region of the *dnaK orf*. PCR product was cloned in-frame with the *gfp* tag on plasmid pBCB1-GE resulting in the construction of plasmid pBCB1-GE 3’ *dnaK*. |
| 1315 | ATAATAGGTACCTTTTTTGTCGTCGTCTTTTACTTCTTTA | R |  |
| 1319 | GTCAAAGTAAACCAATAACTT | F | PCR primers used to confirm integration of the pBCB1-GE 3’*dnaK* into the *dnaK orf* during construction of the single-copy DnaK-GFP fusion in *S. aureus*. |
| 1320 | GTTCTTCTCCTTTGCTAGCC | R |  |
| 1332 | GTATTATTCCAAAAAGGGTTACTCC | F | PCR primer pair used to confirm deletion of the *divIVA orf* in the *S. aureus* *divIVA* mutant |
| 1333 | GCCATTTACATTGTATTGACCA | R |  |
| 1452 | ATTAGGTACCGCACACATTACTGACTTAGGTGG | F | Primer pair containing flanking *Kpn*I restriction sites, used to PCR amplify the *divIVA orf*. PCR product was cloned in-frame with the *gfp* tag on plasmid pBCB1-GE resulting in the construction of plasmid pBCB1-GE IVA700. |
| 1438 | ATTAGGTACCCTTCTTAGTTGTTTCTGAATCATTGG | R |  |
| 1458 | CGGGACAATTAAATGTTTTGGA | F | PCR primer pair flanking the *smc orf* used to confirm disruption of the *smc orf* with Tn917 in the *S. aureus smc* mutant and *smc divIVA* double mutant. |
| 1459 | CCAATTTCGTAAACGGTATCGG | R |  |
| 1460 | GAAAACGGCATTGATTTGTCA | F | PCR primer pair used to confirm disruption of *dnaK* in *B. subtilis* strain SU5 *dnaK:cat*, SU761 *dnaK:cat*. |
| 1461 | CTTTGGCATCGTTGGCTT | R |  |
| 1471 | ATTAGGTACCAAGTGAATCACACTATTGTTGATTC | F | PCR primer pairs containing flanking *Kpn*I restriction sites, used to PCR amplify a fragment containing the *divIVA orf*, RBS, and native promoter. PCR product was cloned in-frame with the *gfp* tag on plasmid pBCB1-GE resulting in the construction of plasmid pBCB1-GE P*_divIVA_* *divIVA-gfp*. |
| 1472 | ATTAGGTACCCTTCTTAGTTGTTTCTGAATCATTGG | R |  |
| 1473 | GATTGGATCCTATGGTGTTGGGTGTTTTCCAATTAGGAATAA | F | PCR primer pairs containing *BamH*I and *Sac*I restriction sites, respectively, used to PCR amplify the *spoIIIE orf* for cloning into plasmid pUT18 and p25N for the construction of pUT18-*spoIIIE* and p25N-*spoIIIE*. |
| 1474 | TTAAGAGCTCAACACCTCGTCATTATTAAGATCTATTAAAACTTGTCTAGG | R |  |
| 1476 | GGTTGACTCTGGCTTTT | R | Sequencing primers to sequence the *spoIIIE orf* cloned into plasmids pUT18-*spoIIIE* and p25N-*spoIIIE*. |
| 1478 | GATGGAATATTATTTTTAATGAT | R |  |
| 1518 | CAAACCATCAACATCGTGAAGT | F |  |
| 1519 | TTAGATGAGAAGCAACCAGAATTACC | F |  |
| 1520 | GGCGCAGAAAAGTTACTTGG | F |  |
| 1479 | GATTGGATCCTATGAGCTGGTTTGATAAATTATTCGGCGAA | F | Primers pairs containing *BamH*I and *Sac*I restriction sites respectively, used to PCR amplify the *ftsK orf* for cloning into plasmid pUT18 and p25N for the construction of pUT18-*ftsK* and p25N-*ftsK*. |
| 1480 | TTAAGAGCTCAAGTTTATGTTACGGAAGCAGATTTAAATAAAGAA | R |  |
| 1481 | GATTGGATCCTATGGTTTATTTAAAATCAATAGATGCC | F | Primers pairs containing *BamH*I and *Kpn*I restriction sites respectively, used to PCR amplify the *smc orf* for cloning into plasmid pUT18 and p25N for the construction of pUT18-*smc* and p25N-*smc*. |
| 1482 | TTAAGGTACCAATTGCTCCTCCTTCAACACA | R |  |
| 1484 | TCACTATGTTTCATTTGATG | R | Sequencing primers to sequence the *smc orf* cloned into plasmids pUT18-*smc* and p25N-*smc*. |
| 1487 | TGTCGATTGATGAACTAGG | F |  |
| 1488 | AGATCTGTACGTTGTTCACT | R |  |
| 1513 | CTCGCAAGGTAGAGTTGATGA | F |  |
| 1515 | TGGTGAGCGTGCATTAACTG | F |  |
| 1516 | CCATTGTTCCTTTACGGTGTGT | R |  |
| 1489 | GATTGGATCCTGTGAGTGAATTGTCAAAAAGTGAAGA | F | Primers pairs containing *BamH*I and *Sac*I restriction sites, respectively, used to PCR amplify the *spoOJ orf* for cloning into plasmid pUT18 and p25N for the construction of pUT18-*spoOJ* and p25N-*spoOJ*. |
| 1490 | TTAAGAGCTCAATTTACCATACCTACGATTTAATTGTTCAATTA | R |  |
| 1492 | TTTCGCTAACCTAAGCA | R | Sequencing primers to sequence the *spoOJ orf* cloned into plasmids pUT18-*spoOJ* and p25N-*spoOJ*. |
| 1517 | GCGGTCATCGAAAATTTACAACG | F |  |
| divIVA_for | AATTAAGGATCCAATGCCTTTTACACCAAATGAA | F | PCR primer pairs containing *BamH*I and *Eco*RI restriction sites, respectively, used to PCR amplify the *divIVA orf* for cloning into plasmid pUT18 and p25N for the construction of pALB46 and pALB50. |
| divIVA_rev | aaaaaagaattcaacttcttagttgtttctgaatcatt | R |  |
| 1565 | ATTAGGATCCCAAGAAAGTTCAAAAATGTGTTTATTC | F | Primer pair containing *BamH*I restriction site, used to PCR amplify ~1.5 kb of upstream flanking region of *divIVA* for subsequent cloning into plasmid pMAD. |
| 1566 | CTTAGTTGTTGGTGTAAAAGGCATTTG | R |  |
| 1567 | TTACACCAACAACTAAGAAGTAAGAATTAAATAAAG | F | Primer pair containing *Bgl*II restriction site, used to PCR amplify ~1.5 kb of downstream flanking region of *divIVA* for subsequent cloning into plasmid pMAD. |
| 1568 | ATTAAGATCTTCTTGTCTTACTTTACTGATTGAG | R |  |
| 1563 | cacgtaatgttgtaactatgaacaat | F | Sequencing primers to sequence the *divIVA* flanking regions to confirm deletion of *divIVA* from chromosome. |
| 1564 | aaatgtatattacgcgtctgtct | R |  |

^a^ Restriction sites are underlined and the nucleotides of the predicted native Shine-Dalgarno sequence are highlighted in bold. ^b^ Primer in the sense direction is designated F and the antisense primer is designated R.

**Supplementary Figures**


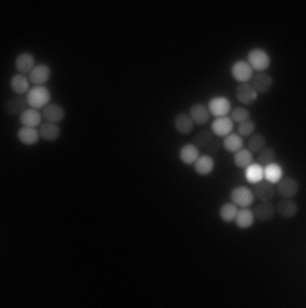


Supplementary Figure 1: *S. aureus* DnaK-GFP localizes uniformly throughout the cytoplasm.

Localization of DnaK-msfGFP in *S. aureus* cells expressing *dnaK-msgfp* from plasmid pLOW (SA307) induced with 1 mM IPTG. Scale bar is 2 µm in length.

A)


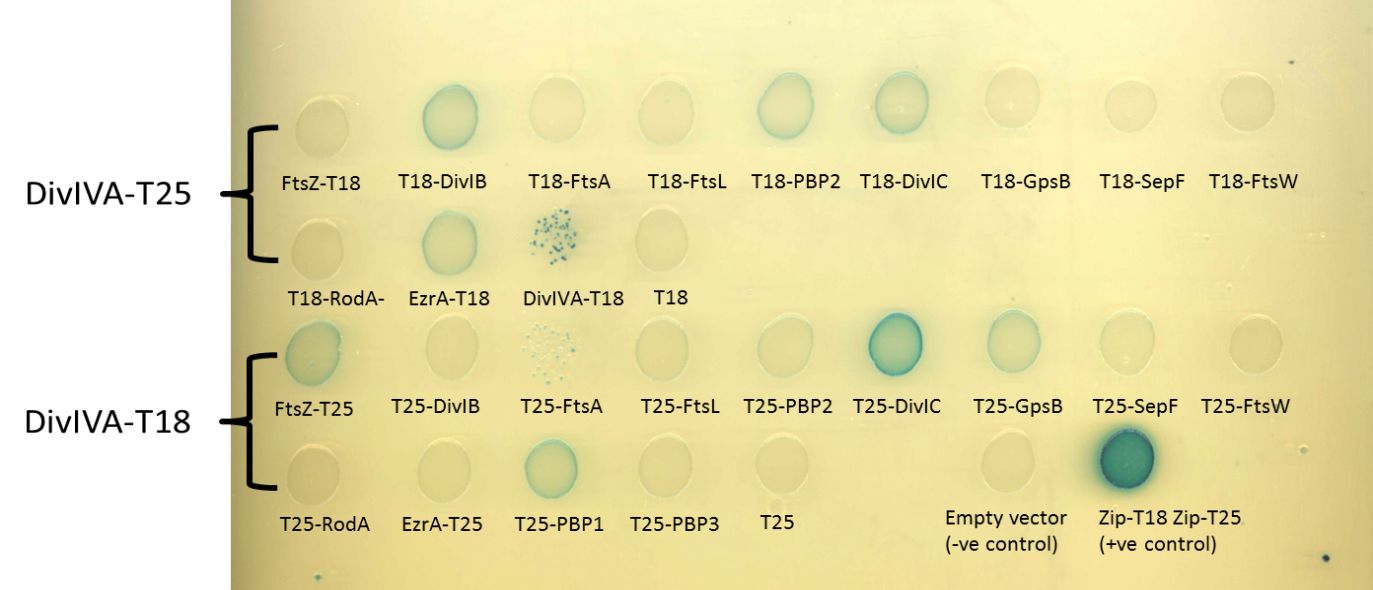


B)

C)

D)

Supp Figure 2: DnaK and DivIVA interact with various divisome components.

Pairwise interactions between DnaK (A), DivIVA (B) and several *S. aureus* divisome proteins were tested using the bacterial-two hybrid assay (Karimova et al., 1998; Steele et al., 2011). Overnight cultures of *E. coli* BTH101 cells containing the respective fusions were drop-plated onto LB agar containing 150 µg/ml X-gal and 0.5 mM IPTG. Cells were grown at 30 °C overnight and then left for 8 hours at room temperature to enhance colour development. Blue-coloured colonies indicate positive interactions between the protein pairs while white colonies indicate no interaction. Cells highlighted with black outlines show pairwise interactions. The red box highlights the positive control cells containing plasmids pKT25-*zip* and pUT18-*zip* (Karimova et al. 1998) as well as cells carrying the control plasmids used to test for non-specific interactions between the *dnaK*, *divIVA* constructs with empty T-18 or T-25 vectors. Both N- and C-terminal fusions of DivIVA and DnaK were tested to attempt to avoid potential functionality issues due to fusion to the T18/T25 fragments.

Quantification of β-galactosidase activity of DnaK (C) and DivIVA (D) BACTH fusions was performed using MUG (4-methylumbelliferyl-β-D-galactopyranoside) as previously described (Steele et al 2011). One MUG unit is defined as the amount of β-galactosidase that catalyses the hydrolysis of 1 pmol of MUG per minute, per ml of culture, per unit of optical density at 600 nm.

Supp Figure 3: The DivIVA-GFP construct is a functional fusion in *S. aureus*.

Percentage anucleate cells in a *S. aureus dnaK::kan* mutant expressing *divIVA-gfp* (SA305) compared to the isogenic *dnaK::kan* mutant (SA210). Cells were grown in BHI broth at 37 °C to mid-exponential phase of growth, stained with fluorescent vancomycin and DAPI and then immobilized on 2% agarose pads for microscopy. Error bars indicate 95% confidence interval obtained from two independent experiments (n= 200 cells). The frequency of anucleate cells in strain SA305 was similar to the SA210 strain (Fisher’s exact test P-value > 0.05). Note that a double *divIVA dnaK::kan* mutant (SA213) shows a two-fold increase in anucleate cells compared to the *dnaK::kan* single mutant (SA210) (Figure 3B) which indicates that the DivIVA-GFP in strain SA305 is functional.

A)

B)

Supp Figure 4: Ectopic expression of *dnaK* rescues the *dnaK::kan* mutant phenotype in *S. aureus*.

A) Cell diameter of *S. aureus dnaK::kan* mutant carrying plasmid pLOW-*dnaK* (SA303) in the presence or absence of 1mM IPTG compared to RN4220 wild-type cells grown without IPTG. n= 200 cells and errors are SEMs derived from two independent experiments.

B) Number of anucleate cells in strain SA303 cells is significantly reduced (Fisher’s exact test P-value < 0.05) in the presence of 1 mM IPTG compared to the *dnaK::kan* mutant (SA210). n= 200 cells and error bars represent 95% CI from two independent experiments. All cells were grown at 37°C in BHI broth to mid-exponential phase of growth with +/- 1 mM IPTG, stained with fluorescent vancomycin and DAPI before being immobilized on 2% agarose pads for microscopy.

CDM

i)

ii)

10^-2^  10^-3^ 10^-4^ 10^-5^ 10^-6^

10^-2^  10^-3^ 10^-4^ 10^-5^ 10^-6^


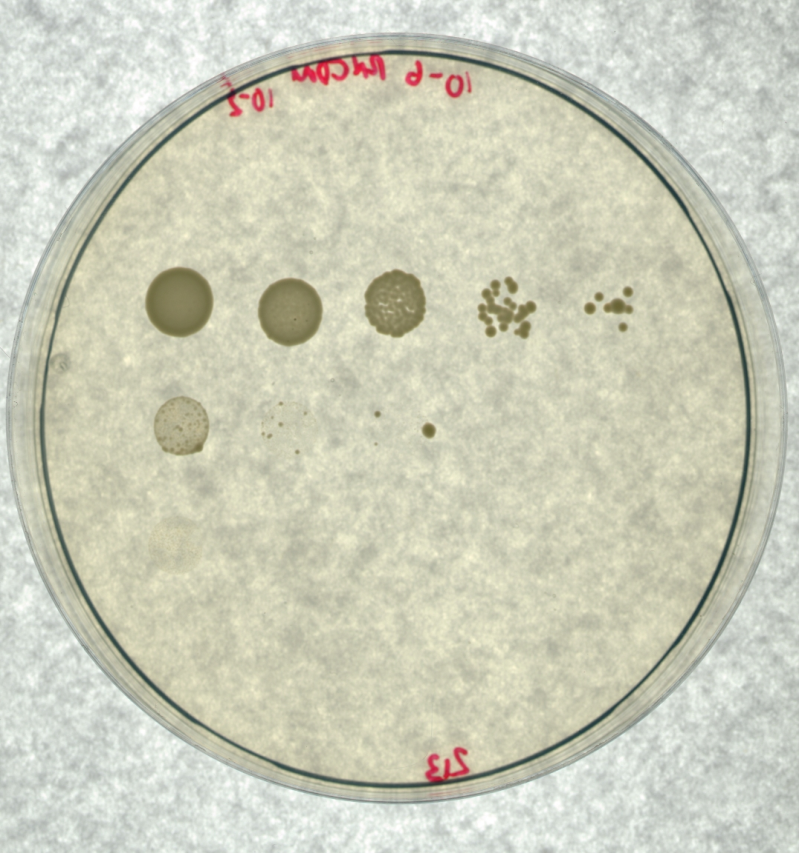

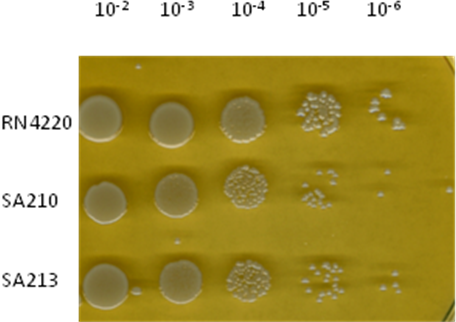


1. ii)

RN4220

*dnaK::kan*

*∆divIVA*

*dnaK::kan*

Supp Figure 5: The ∆*divIVA dnaK::kan* double mutant showed a more severe decreased cell viability in CDM media compared to the *dnaK::kan* single mutant.

RN4220 wild-type, *S. aureus dnaK::kan* (SA210) and *S. aureus ∆divIVA dnaK::kan* double mutant (SA213) were grown to mid-exponential phase of growth at 37°C in BHI (i) or CDM (ii) broth and cell biomass was normalized using OD_600_ values. Serial dilutions (10^-2^ to 10^-6^) of the cultures were prepared in media and 10 µl was drop-plated onto agar plates and incubated at 37°C for 24h.

**
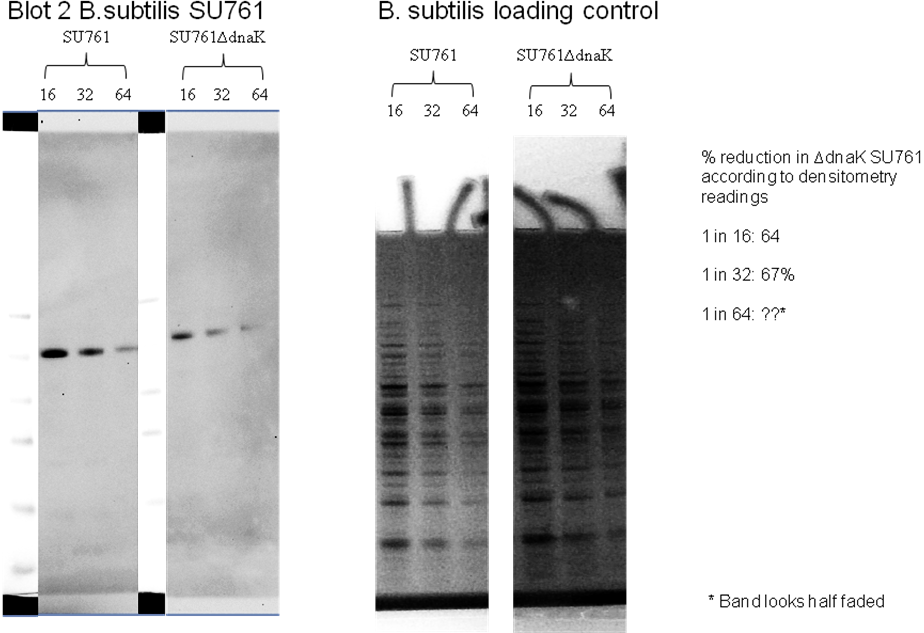

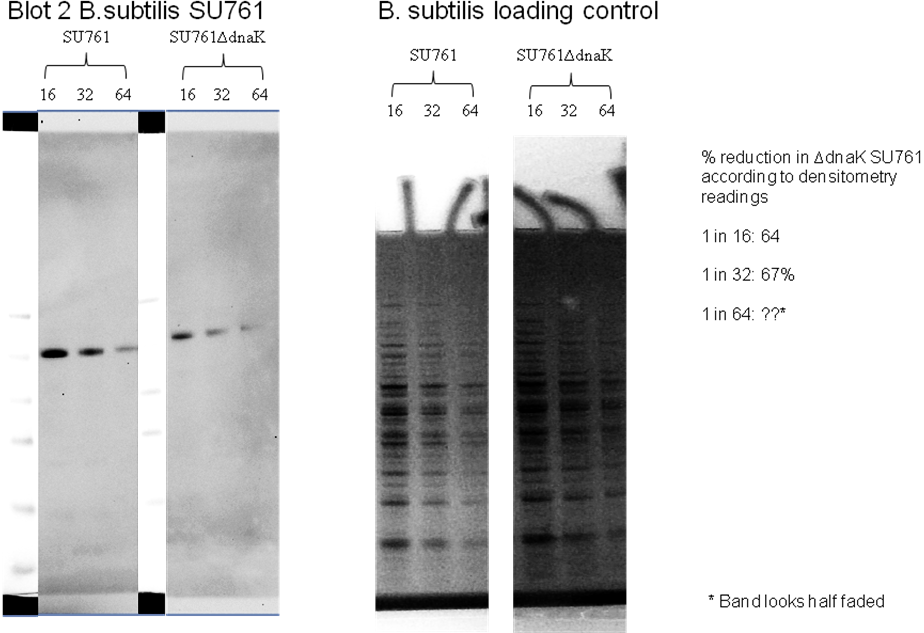
**

49 kDa

1 2 3 4 5 6

A)

**
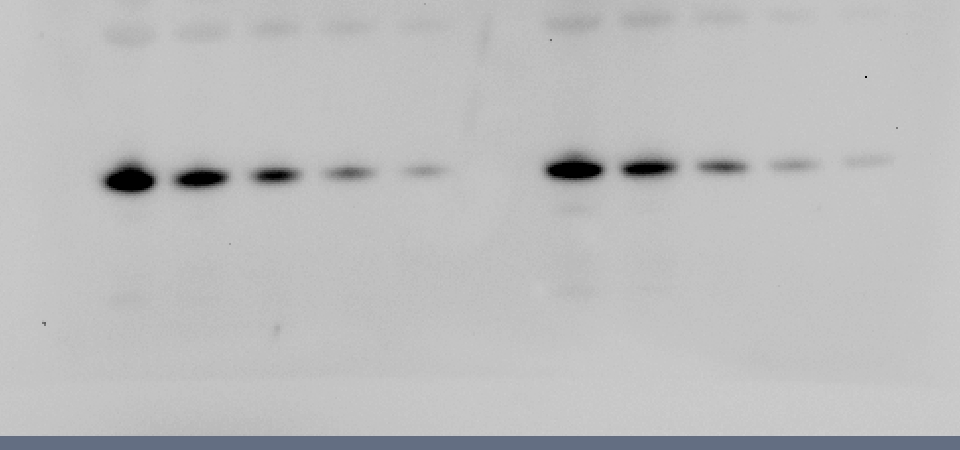

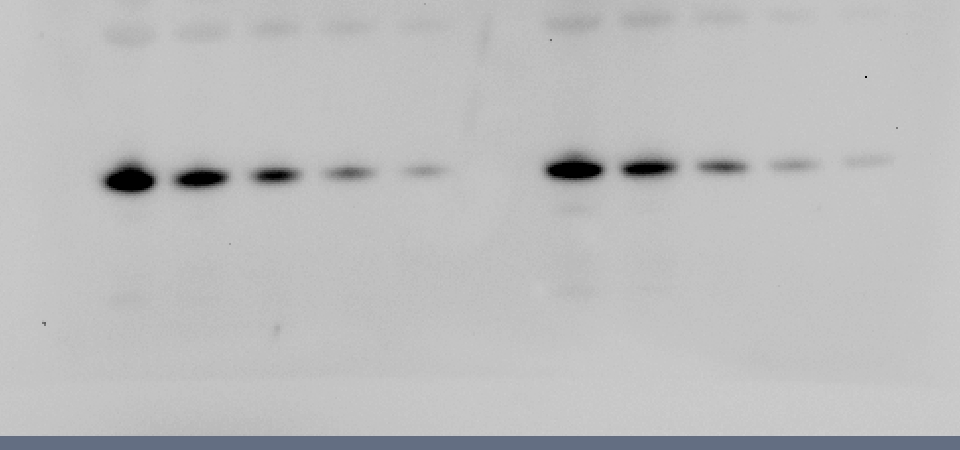
**

B)

23 kDa

1 2 3 4 5 6


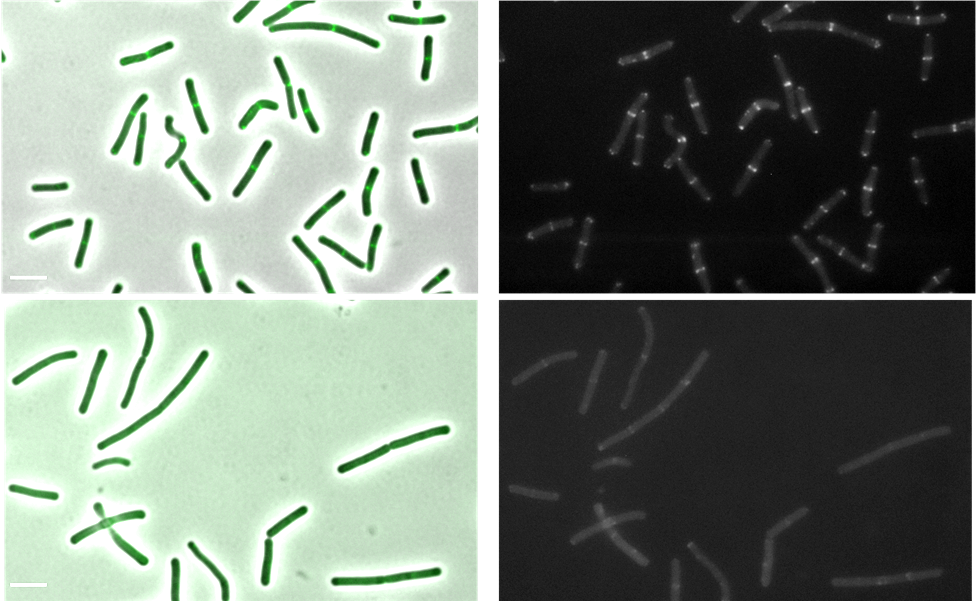

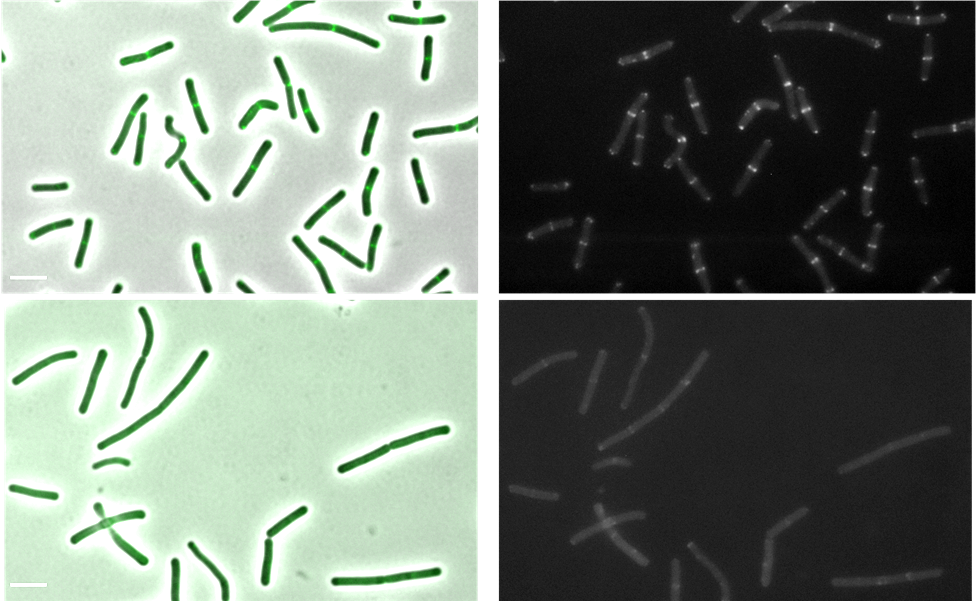


C)

*divIVA-gfp* *dnaK::cat*

*divIVA-gfp*

Supp Figure 6: DivIVA levels are reduced in the *B. subtilis dnaK::cat* insertion mutant.

A) Immuno-blot examining DivIVA-GFP levels in *B. subtilis* cells expressing *divIVA-gfp* (SU761; lanes 1-3) and *B. subtilis* *dnaK::cat* expressing *divIVA-gfp* strain (SU798; lanes 4-6) using anti-GFP antibodies.

B) Immun-oblot examining levels of DivIVA in wild-type *B. subtilis* SU5 (lanes 1-3) and its isogenic *dnaK::cat* mutant (SU797; lanes 4-6) using anti-DivIVA antiserum. Dilution factors of crude lysate used in A & B were: 1 in 16 (lane 1 & 4), 1 in 32 (lane 2 & 5) and 1 in 64 (lane 3 & 6).

C) Fluorescence microscopy of *B. subtilis* cells expressing *divIVA-gfp* (SU761) and its isogenic *dnaK::cat* mutant. *B. subtilis* cells were grown at 37°C to mid-exponential phase of growth in PAB and visualized on 2% agarose pads. Scale bar is 4 µm.


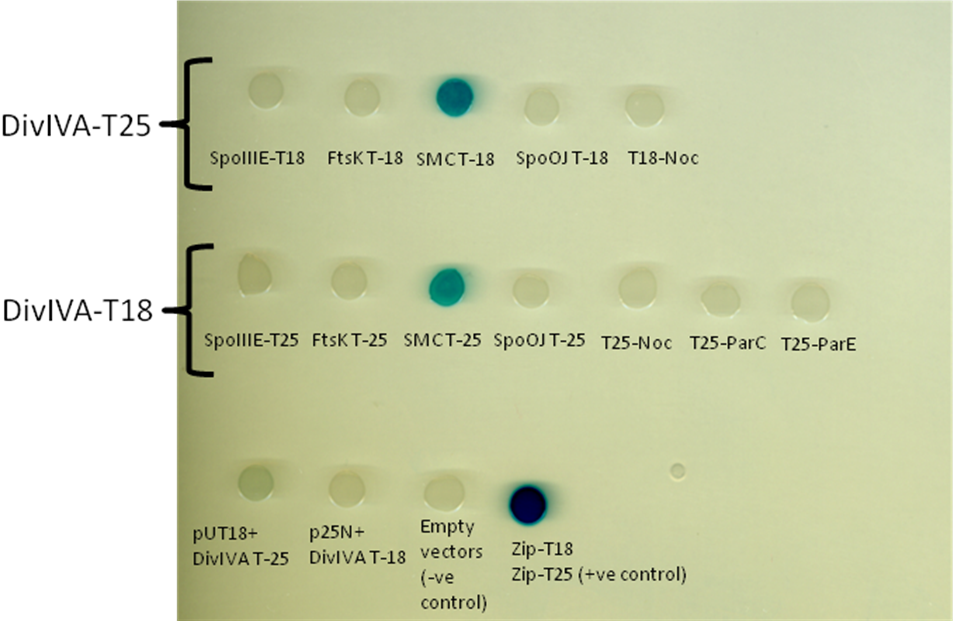
A)

B)

Supp Figure 7: *S. aureus* DivIVA interacts with SMC in a BACTH assay.

A. Overnight cultures of *E. coli* BTH101 cells containing the respective fusions were dropped onto an LB medium plate containing 150µg/ml X-gal and 0.5 mM IPTG. Cells were grown at 30 °C overnight and then left for 8 hours at room temperature. Dark blue colonies (black boxes) indicate a positive interaction. The red box highlights the positive control cells containing plasmids pKT25-*zip* and pUT18-*zip* (Karimova et al. 1998) as well as cells carrying the control plasmids used to test for non-specific interactions between the *divIVA* constructs with empty T-18 or T-25 vectors. Both N- and C-terminal fusions of DivIVA were tested to attempt to avoid potential functionality issues due to fusion to the T18/T25 fragments.

B. Quantification of β-galactosidase activity was performed using MUG (4-methylumbelliferyl-β-D-galactopyranoside) as previously described (Steele et al 2011). One MUG unit is defined as the amount of β-galactosidase that catalyses the hydrolysis of 1 pmol of MUG per minute, per ml of culture, per unit of optical density at 600 nm.

**Supplementary References**

Claessen, D., Emmins, R., Hamoen, L., Daniel, R., Errington, J. & Edwards, D., 2008. Control of the cell elongation-division cycle by shuttling of PBP1 protein in *Bacillus subtilis*. *Molecular Microbiology*, 68(4), pp.1029–1046.

Cooper, E.L., Garcia-Lara, J. & Foster, S., 2009. YsxC, an essential protein in *Staphylococcus aureus* crucial for ribosome assembly/stability. *BMC Microbiology*, 9, doi: 10.1186/1471-2180-9-266.

Karimova, G., Pidoux, J., Ullmann, A. & Ladant, D., 1998. A bacterial two-hybrid system based on a reconstituted signal transduction pathway. *Proceedings of the National Academy of Sciences of the United States of America*, 95(10), pp.5752–5756.

Kreiswirth, B.N., Löfdahl, S., Betley, M., O’Reilly, M., Schlievert, P., Bergdoll, M. & Novick, R., 1983. The toxic shock syndrome exotoxin structural gene is not detectably transmitted by a prophage. *Nature*, 305(5936), pp.709–12.

Landgraf, D., Okumus, B., Chien, P., Baker, T. & Paulsson, J., 2012. Segregation of molecules at cell division reveals native protein localization. *Nature methods*, 9(5), pp.480–2.

Liew, A.T.F., Theis, T., Jensen, S., Garcia-Lara, J., Foster, S., Firth, N., Lewis, P., et al., 2011. A simple plasmid-based system that allows rapid generation of tightly controlled gene expression in *Staphylococcus aureus*. *Microbiology*, 157(3), pp.666–676.

Pereira, P.M., Veiga, H., Jorge, A. & Pinho, M., 2010. Fluorescent reporters for studies of cellular localization of proteins in *Staphylococcus aureus*. *Applied and Environmental Microbiology*, 76(13), pp.4346–4353.

Pinho, M.G. & Errington, J., 2003. Dispersed mode of *Staphylococcus aureus* cell wall synthesis in the absence of the division machinery. *Molecular Microbiology*, 50(3), pp.871-881.

Pinho, M.G. & Errington, J., 2004. A *divIVA* null mutant of *Staphylococcus aureus* undergoes normal cell division. *FEMS Microbiology Letters*, 240(2), pp.145–149.

Schulz, A., Tzsczhaschel, B. & Schumann, W., 1995. Isolation and analysis of mutants of the *dnaK* operon of *Bacillus subtilis*. *Molecular Microbiology*, 15(3), pp.421-429.

Singh, V.K., Utaida, S., Jackson, L., Jayaswal, R., Wilkinson, B. & Chamberlain, N., 2007. Role for *dnaK* locus in tolerance of multiple stresses in *Staphylococcus aureus*. *Microbiology*, 153(9), pp.3162–3173.

Steele, V.R., Bottomley, A., Garcia-Lara, J., Kasturiarachchi, J. & Foster, S., 2011. Multiple essential roles for EzrA in cell division of *Staphylococcus aureus*. *Molecular microbiology*, 80(2), pp.542–55.

Yu, W., Herbert, S., Graumann, P. & Götz, F., 2010. Contribution of SMC (Structural Maintenance of Chromosomes) and spoIIIE to chromosome segregation in staphylococci. *Journal of Bacteriology*, 192(15), pp.4067–4073.
